# *YGL3* Encoding an IPP and DMAPP Synthase Interacts with OsPIL11 to Regulate Chloroplast Development in Rice

**DOI:** 10.1186/s12284-024-00687-y

**Published:** 2024-01-17

**Authors:** Wei Chen, Liqun Tang, Qianlong Li, Yicong Cai, Shakeel Ahmad, Yakun Wang, Shengjia Tang, Naihui Guo, Xiangjin Wei, Shaoqing Tang, Gaoneng Shao, Guiai Jiao, Lihong Xie, Shikai Hu, Zhonghua Sheng, Peisong Hu

**Affiliations:** 1 State Key Laboratory of Rice Biology/Key Laboratory of Rice Biology and Breeding, Ministry of Agriculture/China National Rice improvement Centre, National Rice Research Institute, Hangzhou, 310006 P. R. China; 2 Jiangxi Super-Rice Research and Development Center, Jiangxi Academy of Agricultural Sciences, National Engineering Center for Rice, Nanchang, P. R. China; 3 Key Labora tory of Crop Physiology, Ecology and Genetic Breeding, Research Center of Super Rice Engineering and Technology, Ministry of Education/Collaboration Center for Double-season Rice Modernization Production, Jiangxi Agricultural University, Nanchang, Jiangxi Province 330045 P. R. China

**Keywords:** Isoprenoids, *ygl3*, MEP Pathway, Chloroplast Development, Enzyme Activity, Isopentenyl Diphosphate Content, OsPIL11, *Oryza sativa* L

## Abstract

As the source of isoprenoid precursors, the plastidial methylerythritol phosphate (MEP) pathway plays an essential role in plant development. Here, we report a novel rice (*Oryza sativa* L.) mutant *ygl3* (*yellow-green leaf3*) that exhibits yellow-green leaves and lower photosynthetic efficiency compared to the wild type due to abnormal chloroplast ultrastructure and reduced chlorophyll content. Map-based cloning showed that *YGL3*, one of the major genes involved in the MEP pathway, encodes 4-hydroxy-3-methylbut-2-enyl diphosphate reductase, which is localized in the thylakoid membrane. A single base substitution in *ygl3* plants resulted in lower 4-hydroxy-3-methylbut-2-enyl diphosphate reductase activity and lower contents of isopentenyl diphosphate (IPP) compared to the wild type. The transcript levels of genes involved in the syntheses of chlorophyll and thylakoid membrane proteins were significantly reduced in the *ygl3* mutant compared to the wild type. The phytochrome interacting factor-like gene *OsPIL11* regulated chlorophyll synthesis during the de-etiolation process by directly binding to the promoter of *YGL3* to activate its expression. The findings provides a theoretical basis for understanding the molecular mechanisms by which the MEP pathway regulate chloroplast development in rice.

**Supplementary Information:**

The online version contains supplementary material available at 10.1186/s12284-024-00687-y.

## Background

Chloroplasts are responsible for photosynthesis along with the synthesis of many metabolites including chlorophyll, terpenoids, lipids, amino acids, and hormones in higher plants (Kusumi et al. 2004). As an extremely important pigment in photosynthesis, chlorophyll plays a vital role in harvesting light energy and converting it into chemical energy (Fromme et al. 2003). Therefore, the photosynthetic efficiency of the plant is directly related to the chlorophyll content; higher chlorophyll contents correspond to greater accumulation of photo-assimilates and ultimately higher crop yield (Bansal et al. 1999; Mitchell and Sheehy 2006).

The chlorophyll biosynthesis pathway has been characterized, revealing at least 15 enzymes that are involved in chlorophyll synthesis (Masuda and Fujita 2008). *CHIH, CHLD*, and *CHLI* are the subunits of the Mg-protoporphyrin IX chelatase (Mg-chelatase), which catalyzes the insertion of Mg^2+^ into protoporphyrin IX to form Mg-protoporphyrin IX (MgProto) (Zhang et al. 2006; Tian et al. 2013; Deng et al. 2014). *OsDVR* encodes 3,8-divinyl(proto)chlorophyll(ide), an 8-vinyl reductase, the mutants of which showed yellow-green leaves, reduced chlorophyll contents, and inhibited chloroplast development in comparison to the wild type (Wang et al. 2010, 2013). *OsCAO1* and *OsCAO2*, which encode chlorophyll oxygenase, catalyze the transformation of chlorophyll *a* to chlorophyll *b*. *OsCAO1* and *OsCAO2* synthesize chlorophyll *b* under light and dark conditions, respectively. The mutants of *OsCAO1* and *OsCAO2* showed virescent and green leaves, respectively (Lee et al. 2005; Yang et al. 2016).

Isoprenoids are the most abundant secondary metabolites in plants and play essential roles in photosynthesis, respiration, hormone regulation, biological defense, intracellular signaling, and plant growth and development (Sacchettini and Poulter 1997; Chappell 2002). In living organisms, all isoprenoids are synthesized from two basic five-carbon precursors: IPP and its isomer dimethylallyl diphosphate (DMAPP). Both IPP and DMAPP, which arise from different pathways in different organisms, act as precursors for the synthesis of chlorophyll, carotenoids, hormones, isoprene, and other molecules (Schwender et al. 1996; Eisenreich et al. 1998; Lichtenthaler 1999; Rohmer 1999). In plants, IPP and DMAPP are synthesized through two different pathways: (a) the cytosolic mevalonate (MVA) pathway using acetyl-CoA as a precursor; and (b) the MEP pathway using pyruvate and glyceraldehyde 3-phosphate as substrates. In animals and most eubacteria, IPP and DMAPP are synthesized by the MVA and MEP pathways, respectively. Interestingly, these two pathways are not completely independent, and connections between them have been demonstrated in *rapeseed*, *tobacco*, and *snapdragon* (Kasahara et al. 2002; Bick and Lange 2003; Hemmerlin et al. 2003; Laule et al. 2003).

Phytochrome-interacting factors (PIFs) are a family of eight (PIF1–PIF8) plant-specific basic helix-loop-helix (bHLH) transcription factors (Leivar and Quail 2011; Lee and Choi 2017). PIFs play crucial roles in regulating chlorophyll biosynthesis during seedling de-etiolation. For instance, PIF1 can directly bind to the promoter of *PORC* and activate its expression to regulate the biosynthesis of chlorophyll (Moon et al. 2008). Likewise, *PIF3* negatively regulates chlorophyll biosynthesis by repressing the development of chloroplasts (Stephenson et al. 2009). Nakamura et al. identified six homologs of *Arabidopsis* PIFs known as *Oryza sativa* phytochrome interacting factor-like bHLH genes (*OsPIL11*–*OsPIL16*) have been identified in rice (Nakamura et al. 2007). The *OsPIL13* mutants show pale green leaves with low contents of chlorophyll and chlorophyll-binding protein (Sakuraba et al. 2017).

In this study, we isolated *YGL3* gene via map-based cloning method. The *ygl3* mutant exhibited yellow leaves with significantly lower chlorophyll contents, reduced photosynthetic activity, and abnormal chloroplast structure compared to the wild-type. *YGL3* encoded 4-hydroxy-3-methylbut-2-enyl diphosphate reductase and exhibited enzyme activity. Furthermore, we demonstrated that the OsPIL11 can bind directly to the promoter of *YGL3* to activate its expression and regulate chlorophyll biosynthesis during de-etiolation. The findings provide a solid foundation for understanding the molecular by which the MEP pathway regulates chloroplast development.

## Results

### The *ygl3* Mutant Exhibits Yellow Leaves under Low-temperature Conditions

**Fig. 1:**
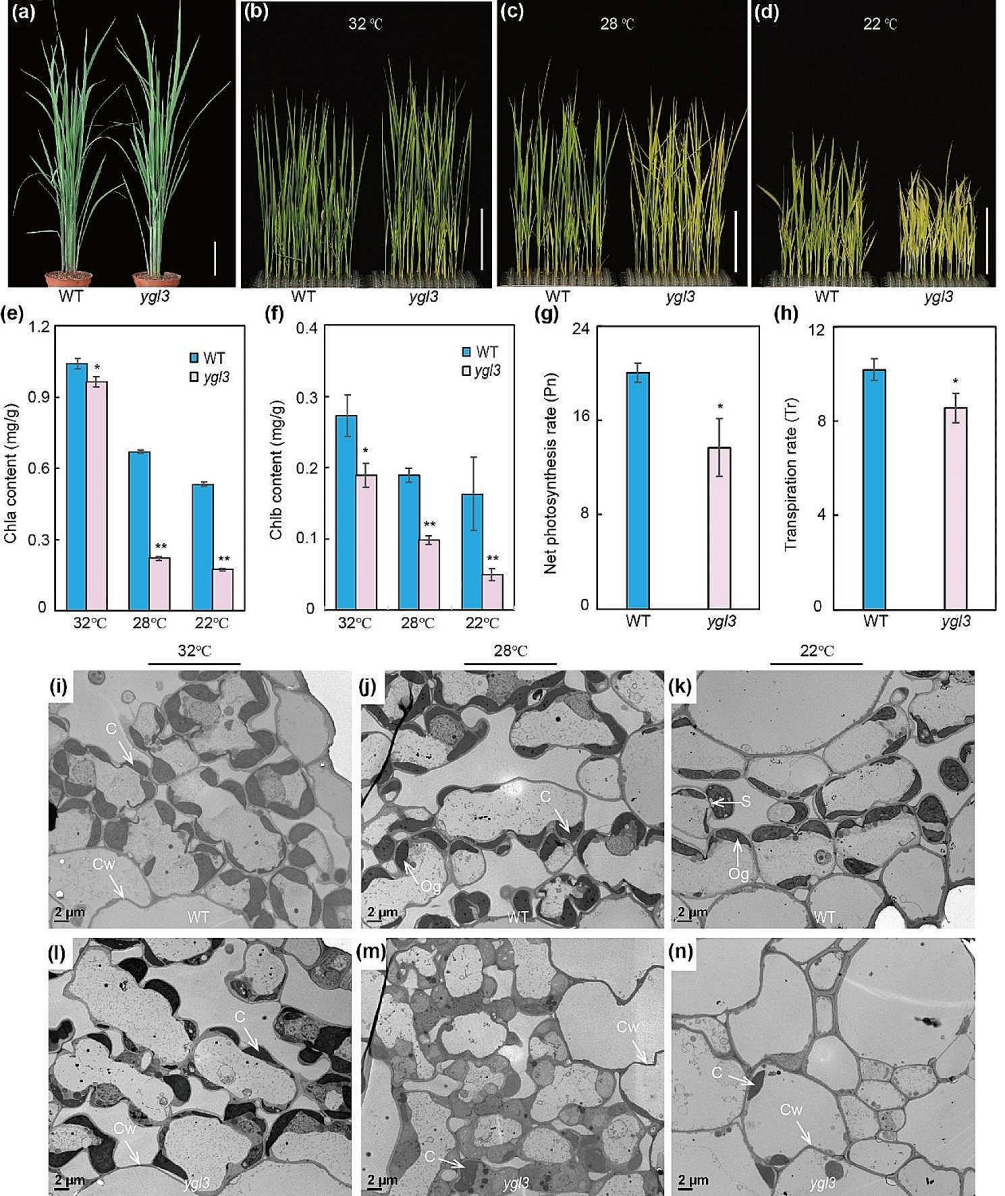
Phenotype of the WT and *ygl3* mutant under low temperature. **(a)** Plants at the tillering stage. Scale bar = 10 cm. **(b–d)** Two-week-old WT and *ygl3* seedlings at 32 °C **(b)**, 28 °C **(c)**, and 22 °C **(d)**. Scale bars = 8 cm. **(e)**, Chlorophyll *a* content in WT and *ygl3* plants at 32 °C, 28 °C, and 22 °C. **(f)** Chlorophyll *b* contents of WT and *ygl3* plants at 32 °C, 28 °C, and 22 °C. **(g and h)** Net photosynthetic rates and transpiration rates of WT and *ygl3* plants in the heading stage. **(i–n)** Ultrastructure of chloroplasts in cells of the leaves of WT (**i**–**k**) and *ygl3* (**l**–**n**) plants at the fourth leaf stage. C, chloroplast; Cw, cell wall; S, starch granule; Og, osmiophilic plastoglobuli. Data are shown as mean ± standard deviation from three biological replicates. Asterisks indicate statistical significance as determined by Student’s *t*-test (**P < 0.01; 0.01 < *P < 0.05)

A plant with yellow-green leaves, provisionally named *ygl3*, was obtained by the ethylmethylsulfone (EMS) mutation of ZHU 1 S (Z1S), a rice thermo-sensitive genic male sterile line that is widely used for rice production in China. The *ygl3* mutant seedlings showed green/yellow-striped leaves after planting in May in Hangzhou, China (Fig. S1a). Under the natural high-temperature conditions in the field, the green/yellow-striped leaves disappeared at the tillering stage (Fig. 1a). To investigate the effect of temperature on *ygl3*, we conducted experiments under different temperatures (22 °C, 28 °C, and 32 °C). Interestingly, the phenotype of the *ygl3* seedlings was similar to that of the wild-type (WT) at 32 °C, whereas green/yellow-striped leaves were observed in *ygl3* at 28 °C (Fig. 1b, c). More severe etiolation was observed in the leaves of the *ygl3* plants at 22 °C (Fig. 1d). Compared to the WT, the plant height and number of tillers were significantly reduced in *ygl3*, whereas the panicle length, grain length, and grain width were not significantly different (Fig. S1b–f).

### The *ygl3* Mutant Showed Defective Chloroplast Development at Low Temperature

To explore the reasons behind the yellow-green leaves in *ygl3*, we evaluated parameters related to chloroplast development. The chlorophyll *a* and *b* contents of *ygl3* mutant were reduced compared to WT at 32 °C, whereas the chlorophyll *a* and *b* content of *ygl3* reduced more significantly at 28 °C and 22 °C, in agreement with the phenotype observed at those temperatures (Fig. 1e, f). Chloroplasts play an important role in the photosynthesis metabolic network. Therefore, we examined the photosynthetic capacity of the WT and *ygl3* under natural conditions. The net photosynthetic rate and transpiration rate were significantly lower in *ygl3* compared to the WT (Fig. 1g, h).

The decreased chlorophyll content was accompanied by changes in the chloroplast ultrastructure. We used transmission electron microscopy (TEM) to examine the chloroplast structure in the WT and *ygl3* at the fourth leaf stage. The chloroplasts, grana, and lamella were well developed and well structure in both the WT and *ygl3* at 32 °C (Fig. 1i, l; Fig. S2a, d). In contrast, at 28 °C, *ygl3* exhibited fewer chloroplasts and more immature chloroplasts compared to the WT, and we also observed a vestigial thylakoid membrane ultrastructure in *ygl3* (Fig. 1j, m; Fig. S2b, e). Interestingly, a few normal chloroplasts were also observed in *ygl3* at 28 °C, corresponding to the yellow/green-striped leaves at that temperature (Fig. 1c, j, m). Moreover, at 22 °C, the chloroplasts were barely visible in the *ygl3* mesophyll cells (Fig. 1k, n; Fig. S2c, f). These results indicate that *YGL3* was involved in chloroplast development at low temperature.

### Map-Based Cloning of *YGL3*

**Fig. 2:**
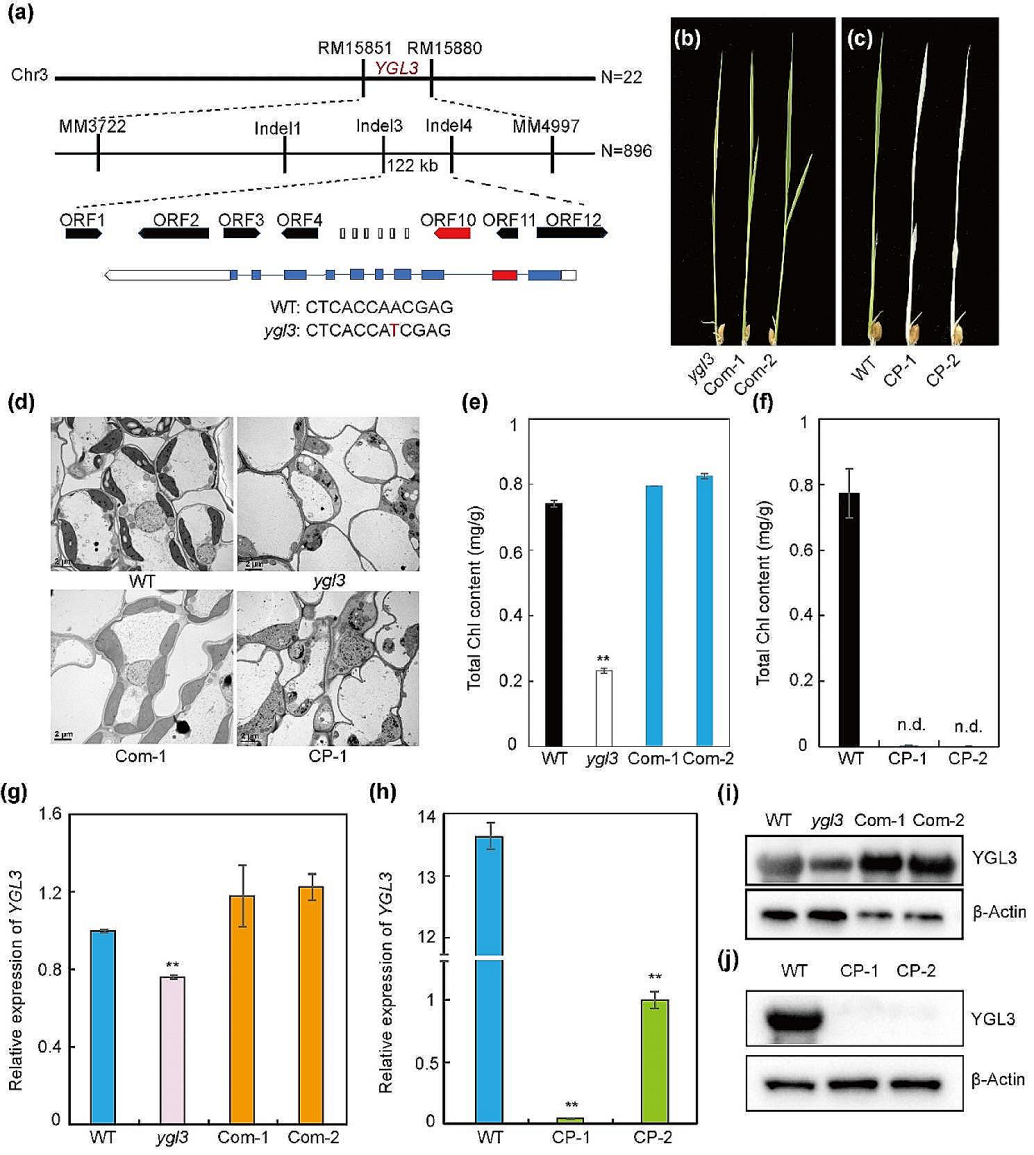
Map-based cloning of *YGL3*. **(a)** Mapping of the *YGL3* locus in a 122-kb region on Chromosome 3 (Chr3). **(b)** Phenotypic characteristics of complementation plants (Com-1 and Com-2) of *ygl3* at 28℃. Scale bar = 2 cm. **(c)** Phenotypic characteristics of knockout plants (CP-1 and CP-2) of *YGL3* at 28℃. Scale bar = 2 cm. **(d)** Chloroplast ultrastructures of WT, *ygl3*, complementation (Com-1), and knockout transgenic plants (CP-1) at 28℃. **(e and f)** Chlorophyll contents of WT, *ygl3*, complementation plants, and knockout plants. **(g and h)** Transcriptional levels of *YGL3* in complementation and knockout transgenic plants. The RNA was extracted from two-week-old plants. The rice *Actin* gene was used as the internal control. **(i and j)** Immunoblot analysis of YGL3 protein in WT, *ygl3*, and transgenic plants at 28℃. Total protein was extracted from two-week-old plants. β-Actin was used as a loading control. Data are shown as mean ± standard deviation from three biological replicates. Asterisks indicate statistical significance as determined by Student’s *t*-test (**P < 0.01; 0.01 < *P < 0.05)

To analyze the genetic characteristics of *YGL3*, the *ygl3* plants were crossed with NIP (*Nipponbare*, *japonica* rice), D50 (tropical *japonica* rice), and 02428 (*japonica* rice). The leaves in all F_1_ progenies were similar to those in the WT. In the F_2_ population, the ratio of green to yellow-green leaves was 3:1, consistent with the Mendelian monogenic ratio; this indicates that the yellow-green leaves are controlled by a monogenic recessive gene (Table S1). Using linkage analysis, we found that *ygl3* was located between the SSR markers RM15851 and RM15880 on chromosome 3. Based on 896 plants with yellow-green leaves from the F_2_ population of *ygl3*/NIP, the *YGL3* locus was further narrowed to a 122-kb region between InDel markers 3 and 4. Within the region, we predicted 12 ORFs (Open Reading Frames) (Table S2). Sequencing analysis revealed that a single nucleotide transversion (A→T) occurred in the second exon of ORF10 (*LOC_Os03g52170*), resulting in the replacement of asparagine with isoleucine and changes in the three-dimensional structure of the protein (Fig. 2a; Fig. S3).

To further validate the role of *YGL3* in chlorophyll biosynthesis, we performed gene complementation and knockout experiments using transgenic technology. In a gene complementation assay, we amplified the *YGL3* coding region along with the 2-kb upstream and 1.5-kb downstream regions and then cloned them into the binary vector pCAMBIA1300. For the knockout experiment, we constructed a vector carrying the machinery of the CRISPR/Cas9 system. The complementation and CRISPR/Cas9 vectors were introduced into *ygl3* and *Nipponbare*, respectively. As expected, the yellow-green phenotype of *ygl3* was reverted into the normal phenotype in the complementation plants (COM-1 and COM-2). The pigment contents and chloroplast ultrastructure were also restored (Fig. 2b, d, e). Moreover, the mRNA and protein contents of *YGL3* were also returned to normal (Fig. 2g, i). On the other hand, two homozygous plants of knockout *YGL3* gene under NIP background showed albino seedlings and died at the third leaf stage (Fig. 2c; Fig. S4). In addition, the knockout lines exhibited lower chlorophyll contents and abnormal chloroplast ultrastructure compared with the WT (Fig. 2c, d, f). As in the *ygl3* mutant, the transcription levels and protein levels were significantly lower in the knockout mutants compared to the WT (Fig. 2h, j). Consequently, ORF10 (*LOC_Os03g52170*) clearly corresponded to *YGL3*.

The protein sequence and phylogenetic analysis revealed that *YGL3* was highly conserved and encoded a 4-hydroxy-3-methylbut-2-enyl diphosphate reductase. Homologs of *YGL3* are found in other plants including *A. thaliana, Zea mays*, *Sorghum bicolor*, *Triticum urartu*, and *Setaria italica* (Fig. S5; Fig. S6).

### *YGL3* is Ubiquitously Expressed, and its Protein Localizes in the Chloroplast

**Fig. 3:**
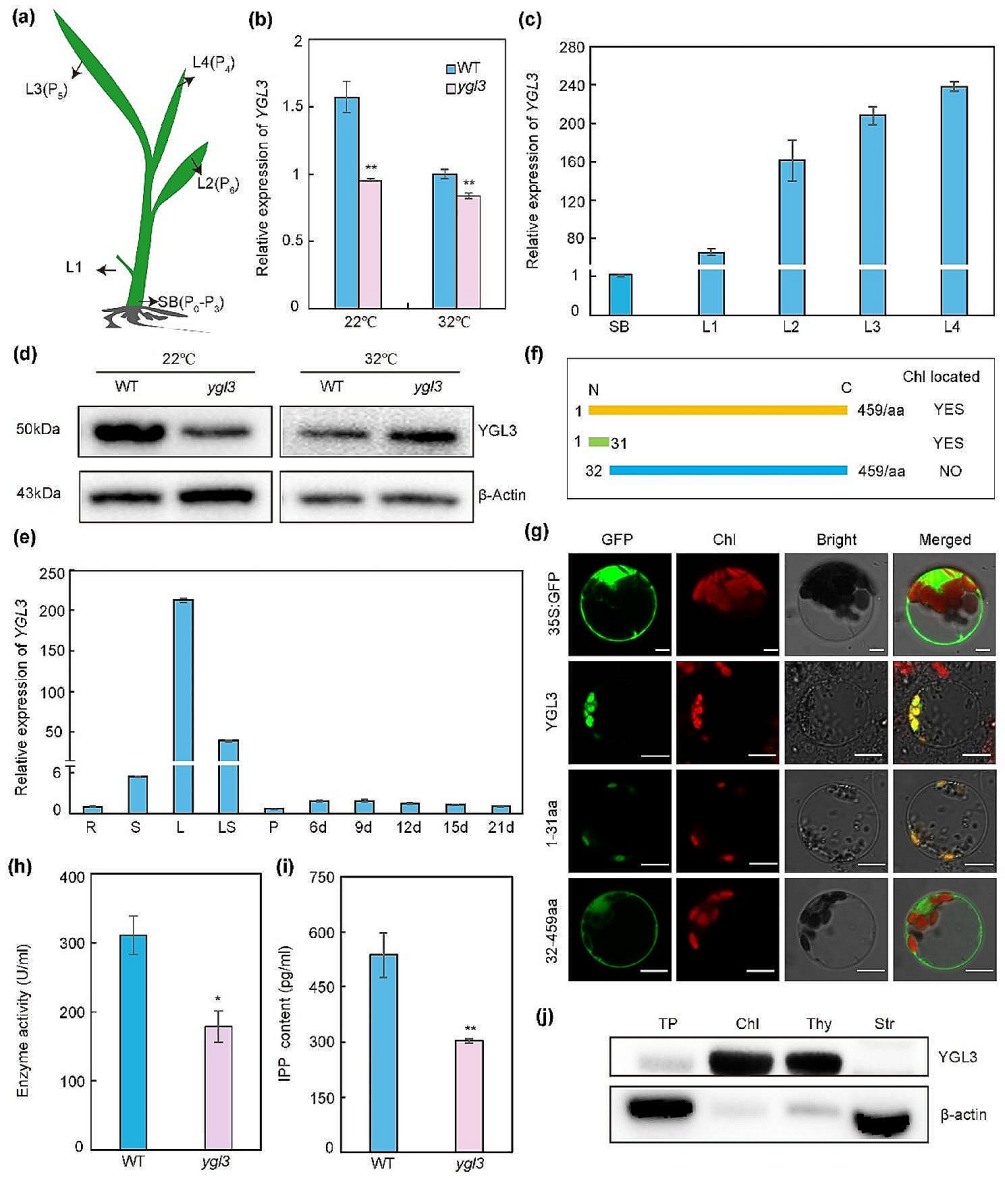
Expression, subcellular localization, and enzyme activity of YGL3. **(a)** Schematic diagram of a rice seedling at the fourth leaf stage. SB, shoot base, corresponding to the stages P0–P3 of leaf development; L1–L4, the first leaf to fourth leaf. **(b)** Expression levels of *YGL3* in WT and *ygl3* plants at 32 °C and 22 °C. The RNA was extracted from two-week-old plants. The rice *Actin* gene was used as the internal control. **(c)** Expression of *YGL3* in WT plants at the fourth leaf stage. **(d)** Protein abundance of YGL3 in WT and *ygl3* plants at 32 °C and 22 °C. Total protein was extracted from leaves of two-week-old plants. β-Actin was used as a loading control. **(e)** qRT-PCR analysis of the expression levels of YGL3 in various tissues. R, root; S, stem; L, leaf; LS, leaf sheath; P, panicle; 6 d, 9 d, 12 d, 15 d, and 21 d correspond to the developing endosperm at 6, 9, 12, 15, and 21 d after fertilization, respectively. **(f)** Schematic illustration of the different lengths of YGL3 protein. **(g)** Subcellular location of YGL3, YGL3^1–31aa^, and YGL3^32–459aa^ in rice protoplasts. GFP, green fluorescence; Red, chloroplast autofluorescence. Scale bars = 5 μm. **(h)** Enzyme activity of 4-hydroxy-3-methylbut-2-enyl diphosphate reductase in WT and *ygl3* plants. Total protein was extracted from two-week-old plants. **(i)** IPP contents of WT and *ygl3* plants. Total protein was extracted from two-week-old plants. **(j)** Immunoblot analysis of YGL3 protein. Protein samples were extracted from WT. β‐Actin was used as a loading control. TP, total protein; Chl, chloroplast; Thy, thylakoid membrane fraction; Str, stroma fraction. Data are shown as mean ± standard deviation from three biological replicates. Asterisks indicate statistical significance as determined by Student’s t-test (**P < 0.01; 0.01 < *P < 0.05)

The morphological development of rice leaves can be divided into six stages: P0 (leaf founder); P1 (youngest primordium); P2, P3, and P4 (early leaf and chloroplast development); and P5 and P6 (mature leaf) (van Campen et al. 2016). *YGL3* is highly expressed in younger leaves such as L4, which corresponds to the P4 stage of leaf development (Fig. 3a, c). The temporal and spatial patterns of *YGL3* expression were analyzed based on the data available online from NCBI (https://www.ncbi.nlm.nih.gov/). The results indicate that *YGL3* is widely expressed in rice. These data were further validated by quantitative real-time polymerase chain reaction (qRT-PCR). The *YGL3* is constitutively expressed, with the highest expressions observed in the leaf and leaf sheath followed by the root, stem, and panicle, and developing grains (Fig. 3e). We further investigated the expression of *YGL3* by constructing a vector with the GUS reporter gene driven by the *YGL3* promoter and transformed it into rice. Histochemical analysis showed that *YGL3* was expressed constitutively; GUS activity accumulated more in leaf, in agreement with the qRT-PCR results (Fig. S7a–d). The expression of *YGL3* was lower in *ygl3* than in the WT at both 22 and 32 °C (Fig. 3b). Interestingly, the content of YGL3 protein in *ygl3* was higher than that in the WT at 32 °C (Fig. 3d). Based on these data, we speculated that a feedback mechanism could be present at 32 °C.

An analysis of the subcellular localization of YGL3 using Cell-PLoc 2.0 (http://www.csbio.sjtu.edu.cn/bioinf/Cell-PLoc-2/) suggested that YGL3 is located in the chloroplasts, and that the 1–30 amino acids are chloroplast signaling peptides. To validate this prediction and identify the subcellular localization of YGL3, we constructed a vector to deliver the *35 S*: YGL3: GFP and transiently expressed in rice protoplasts. As expected, the signal of GFP was observed in the chloroplasts (Fig. 3f, g). To verify the chloroplast-targeting signaling peptides in YGL3, YGL3^1–31aa^-GFP and YGL3^32–459aa^-GFP were constructed and transiently expressed in rice protoplasts. The GFP signals were produced in chloroplasts by YGL3 ^1–31aa^-GFP, whereas the YGL3^32–459aa^-GFP-truncated protein did not show any signal (Fig. 3f, g). To further determine the precise localization of YGL3, we performed immunoblot analysis. We extracted total protein, chloroplast, thylakoid membrane, and stromal fractions from the WT. YGL3 was present in all fractions except the stromal fraction (Fig. 3j), suggesting that YGL3 is a thylakoid membrane localization protein, and that the 1-31aa segment is required for chloroplast targeting.

### Loss of *YGL3* Function Results in Decreased 4-hydroxy-3-methylbut-2-enyl Diphosphate Reductase Activity

The *ygl3* mutant exhibited mutation in the LTYB domain, which is involved in the activity of 4-hydroxy-3-methylbut-2-enyl diphosphate reductase (Fig. S6a). Thus, we speculated that this mutation affected the enzymatic activity of 4-hydroxy-3-methylbut-2-enyl diphosphate reductase. Indeed, the enzyme activity of 4-hydroxy-3-methylbut-2-enyl diphosphate reductase was dramatically reduced in *ygl3* compared to the WT (Fig. 3h). Eisenreich et al. reported that 4-hydroxy-3-methylbut-2-enyl diphosphate reductase catalyzes the transformation of 4-hydroxy-3-methyl-but-2-enyl pyrophosphate (HMBPP) into IPP in the last step of the MEP pathway (Eisenreich et al. 1998). To verify our results, we measured the contents of IPP. The IPP contents were significantly lower in *ygl3* compared with the WT (Fig. 3i). Similarly, as expected, the 4-hydroxy-3-methylbut-2-enyl diphosphate reductase activity and IPP contents were dramatically reduced in the knockout mutant plants and increased in complementary transgenic plants compared to the WT (Fig. S8a–c). These results demonstrate that YGL3 exhibited 4-hydroxy-3-methylbut-2-enyl diphosphate reductase activity, and that the mutation in the LTYB domain compromised the enzymatic activity.

### *YGL3* is Required for the Chloroplast Development in Rice

**Fig. 4:**
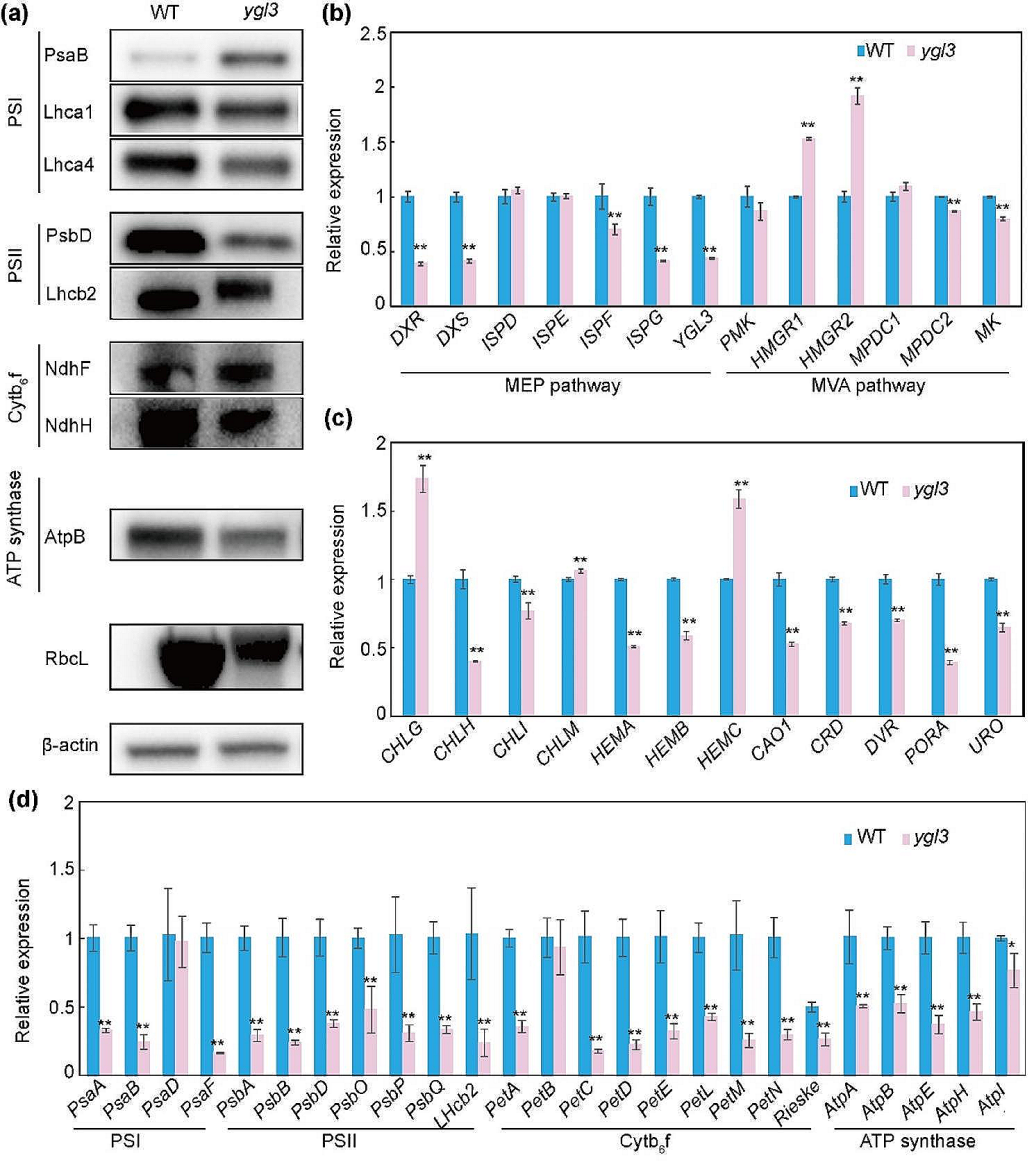
Expression and immunoblot analysis of genes in WT and *ygl3* seedlings grown at 28 °C. **(a)** Levels of thylakoid membrane proteins in WT and *ygl3* plants at the seedling stage. β-Actin was used as a loading control. **(b)** Relative expressions of genes involved in the MEP and MVA pathways in WT and *ygl3* plants. **(c)** Relative expressions of genes involved in chlorophyll biosynthesis. **(d)** Relative expressions of thylakoid membrane protein genes in WT and *ygl3* plants. Data shown are mean ± standard deviation from three biological replicates. Asterisks indicate statistical significance as determined by Student’s *t*-test (**P < 0.01; 0.01 < *P < 0.05)

The thylakoid membrane is an important component of chloroplasts where the photosynthetic light reaction occurs. The protein complexes of the thylakoid membrane are composed of photosystem I (PSI), photosystem II (PSII), cytochrome b6f complex, and adenosine 5-triphosphate (ATP) synthase (Hartings et al. 2017). Considering the abnormal lamellar structure of thylakoid in *ygl3*, we detected the levels of major thylakoid membrane protein complexes in the WT and *ygl3* by immunoblot analysis. We observed significant differences in the core subunits of PSI and PSII between the WT and *ygl3*. For instance, PsaB and PsbD were significantly increased and decreased in *ygl3* compared to the WT, respectively (Fig. 4a). Moreover, the chlorophyll *a* and *b*-binding proteins involved in light-harvesting complexes were significantly reduced in *ygl3* compared to the WT (Fig. 4a). Furthermore, the contents of NdhF (the NAD(P)H dehydrogenase subunit), RbcL (the RuBisco large subunit), and the AtpB (Beta subunit of ATP synthase) were significantly reduced in *ygl3* compared to the WT (Fig. 4a). We also evaluated the transcript levels of thylakoid membrane proteins by qRT-PCR. The transcript levels of all thylakoid membrane proteins except for *PsaD* and *PetB* were notably downregulated in *ygl3* compared to the WT (Fig. 4d). We also determined the transcript levels of genes associated with chlorophyll synthesis. These genes (such as *CHLH, CHLI, HEMA, HEMB, CAO1, CRD, DVR, PORA*, and *URO*) were significantly downregulated in *ygl3* compared to the WT (Fig. 4c). Collectively, these results suggest that *YGL3* plays a crucial role in chloroplast development.

### The Mutation of *YGL3* Disturbs the MEP and MVA Pathways

YGL3, which is the final rate-limiting enzyme in the MEP pathway, is homologous to ISPH in *Arabidopsis*. To explore the effects of *YGL3* mutation on the MEP pathway, we examined the transcript levels of MEP pathway-related homologous genes (*DXS*, *DXR*, *ISPD*, *ISPE*, *ISPF*, *ISPG*, and *YGL3*) in the WT and *ygl3* using qRT-PCR. The expressions of *DXR*, *DXS*, *ISPF*, *ISPG*, and *YGL3* genes were dramatically decreased in *ygl3* compared to the WT (Fig. 4b). In higher plants, the MEP and MVA pathways are not completely independent of each other. Hence, we detected the mRNA levels of genes associated with the MVA pathway (*HMGR1*, *HMGR2*, *MK*, *PMK*, *MPDC1*, and *MPDC2*). The transcription levels of *HMGR1*, *HMGR2*, *MPDC1, MPDC2*, and *MK* were remarkably different between the *ygl3* mutant and the WT (Fig. 4b). These results indicate that the mutation of *YGL3* disturbed the equilibrium of both the MEP and MVA pathways.

### OsPIL11 Activated the Expression of *YGL3* by Directly Binding to its Promoter

**Fig. 5:**
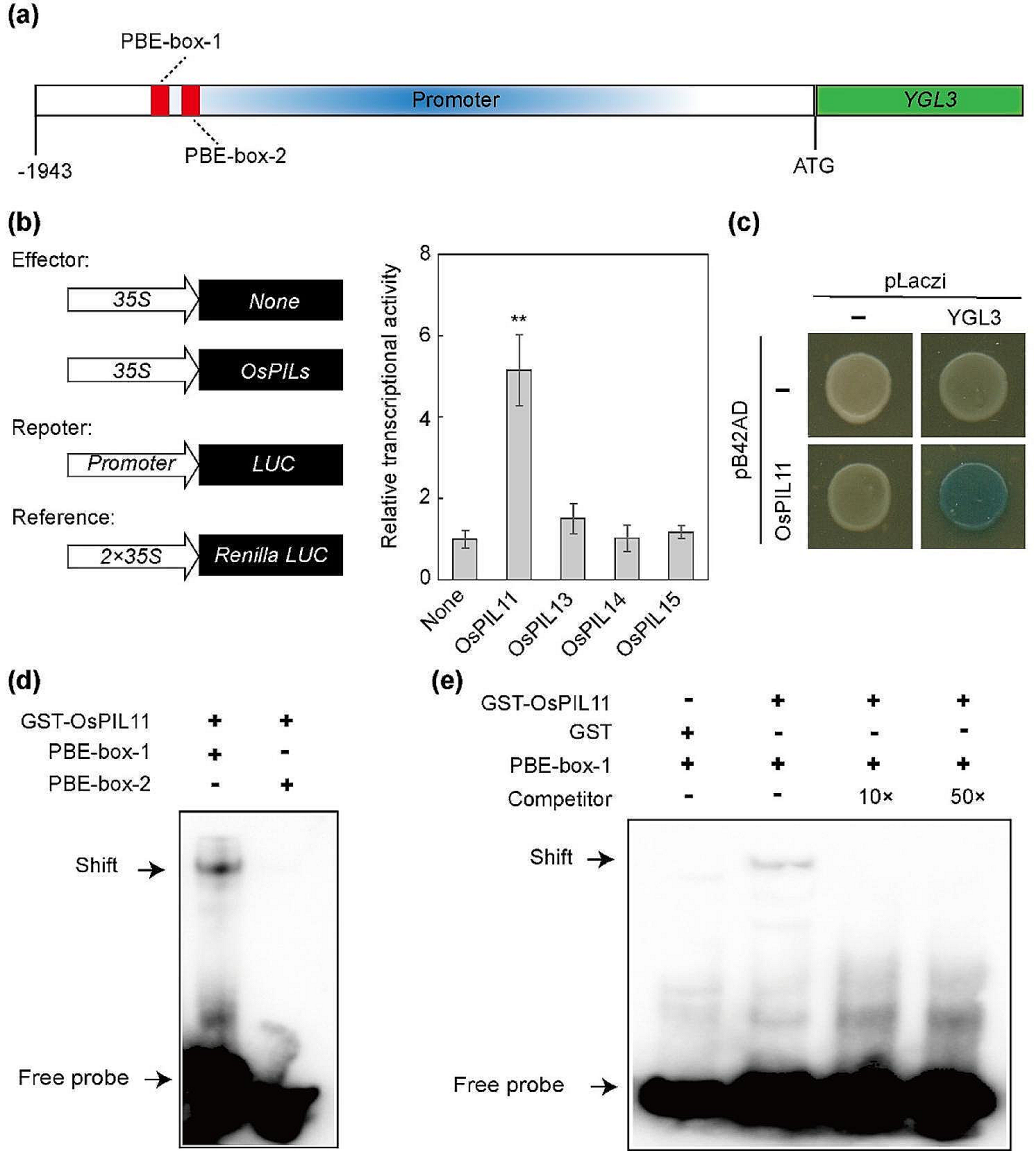
OsPIL11 directly binds to the *YGL3* promoter. **(a)** Schematic showing the localization of the PBE-box in the *YGL3* promoter. **(b)** Transcriptional activation activities of OsPILs on *YGL3* promoters in rice protoplasts. Relative LUC activity was calculated as LUC/REN. (**c**) Yeast one-hybrid assay showed that OsPIL11 can bind to the *YGL3* promoter. **(d and e)** EMSA assay showed that OsPIL11 can directly bind to the promoter of *YGL3*. Ten- and 20-fold excesses of non-labeled probes were used for competition. Asterisks indicate statistical significance as determined by Student’s t-test (**P < 0.01; 0.01 < *P < 0.05)

**Fig. 6:**
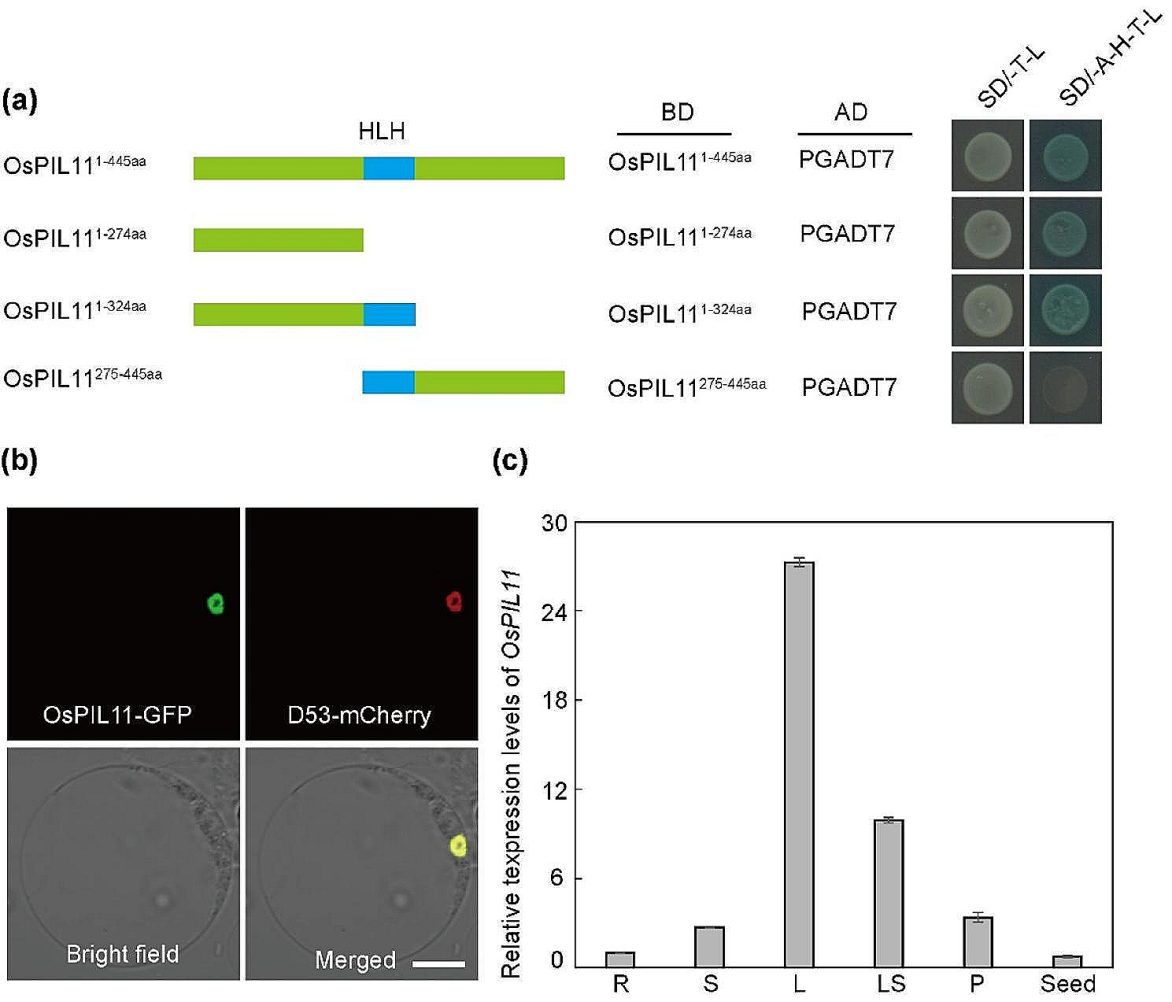
*OsPIL11* facilitates the greening of etiolated seedlings. **(a)** Phenotypes of WT and *ygl3* plants after light irradiation for 12 h at 28 °C. Ten-day-old etiolated seedlings were exposed to light. **(b)** Chlorophyll synthesis rate of 10-day-old etiolated seedlings (WT and *ygl3*) after light irradiation for 24 h at 28 °C. **(c)** Phenotypes of SN265 and *OE*-*OsPIL11* plants after light irradiation for 12 h at 28 °C. Ten-day-old etiolated seedlings were exposed to light. Scale bar = 2 cm. **(d and e)** Chloroplast ultrastructures in SN265 and *OE-OsPIL11* plants. **(f)** Chlorophyll contents of the SN265 and *OE-OsPIL11* plants. Etiolated seedlings were grown in the darkness for 10 d at 28 °C and then exposed to light for 24 h. **(g)** Relative expressions of *YGL3* and *OsPIL11* in SN265 and *OE-OsPIL11* plants based on qRT**-**PCR analysis. RNA was extracted from seedlings grown in the darkness for 10 d at 28 °C. **(h)** Activity of 4-hydroxy-3-methylbut-2-enyl diphosphate reductase in SN265 and *OE-OsPIL11* plants. **(i)** IPP contents in SN265 and *OE-OsPIL11* plants. **(j)** Transcription levels of thylakoid membrane genes in SN265 and *OE-OsPIL11* plants. **(k)** Transcription levels of chlorophyll synthesis genes in SN265 and *OE-OsPIL11* plants. **(l)** Proposed model for the regulation of chlorophyll synthesis by *YGL3*. *YGL3* encodes a 4-hydroxy-3-methylbut-2-enyl diphosphate reductase that catalyzes the transformation of HMBPP to IPP and DMAPP, which are the precursors of chlorophyll synthesis. OsPIL11 directly activates the expression of *YGL3* by binding to its promoter, increasing the contents of IPP and DMAPP to synthesize more chlorophyll. G3P, glyceraldehyde 3-phosphate; HMBPP, 4-hydroxy-3-methylbut-2-enyl diphosphate; IPP, isopentenyl diphosphate; DMAPP, dimethylallyl diphosphate. Asterisks indicate statistical significance as determined by Student’s *t*-test (**P < 0.01; 0.01 < *P < 0.05)

To identify the key transcription regulators of *YGL3*, we analyzed the *cis*-elements of the *YGL3* promoter. Two putative PBE-box (CACATG) motifs were located in the promoter region (Fig. 5a). The PBE-box is a *cis*-element that is recognized by bHLH transcription factors (Zhang et al. 2013). PIF proteins belong to a subset of bHLH transcript factors that play crucial roles in plant development, including chlorophyll synthesis, circadian clock, photomorphogenesis, and flowering (Huq et al. 2004; Leivar et al., 2014). Nakamura et al. identified the homologous genes of *AtPIFs* in rice and designated them as *OsPILs* (*OsPIL11*–*OsPIL16*). We used luciferase transient transcriptional activity assay to screen the upstream transcription factors of *YGL3*. We selected four genes (*OsPIL11*, *OsPIL13*, *OsPIL14*, and *OsPIL15*) as activators for analysis. *OsPIL11* significantly activated the transcription of *YGL3*, whereas the negative control (None vector), OsPIL13, OsPIL14, and OsPIL15 did not (Fig. 5b). We examined the direct binding of the *YGL3* promoter by OsPIL11 by yeast one-hybrid assay, indicating that OsPIL11 specifically bound to the *YGL3* promoter (Fig. 5c). Subsequently, we tested the direct binding of OsPIL11 in vitro by electrophoretic mobility shift assay (EMSA) using both PBE-box-1 and PBE-box-2. GST-OsPIL11 but not GST bound to PBE-box-1 to retard the migration of it (Fig. 5d). However, a weak protein-probe complex shift band was observed upon adding the competitive probe (Fig. 5e). Considering the critical role of OsPIL11 in activating the transcription of *YGL3*, we examined the transcription levels of *YGL3* in *OE-OsPIL11* transgenic plants. The transcript levels of *YGL3* were remarkably increased in *OE-OsPIL11* plants compared to the WT (Fig. 6g). These results confirm that OsPIL11 bound directly to the promoter of *YGL3* and activated its expression.

**Fig. 7:**
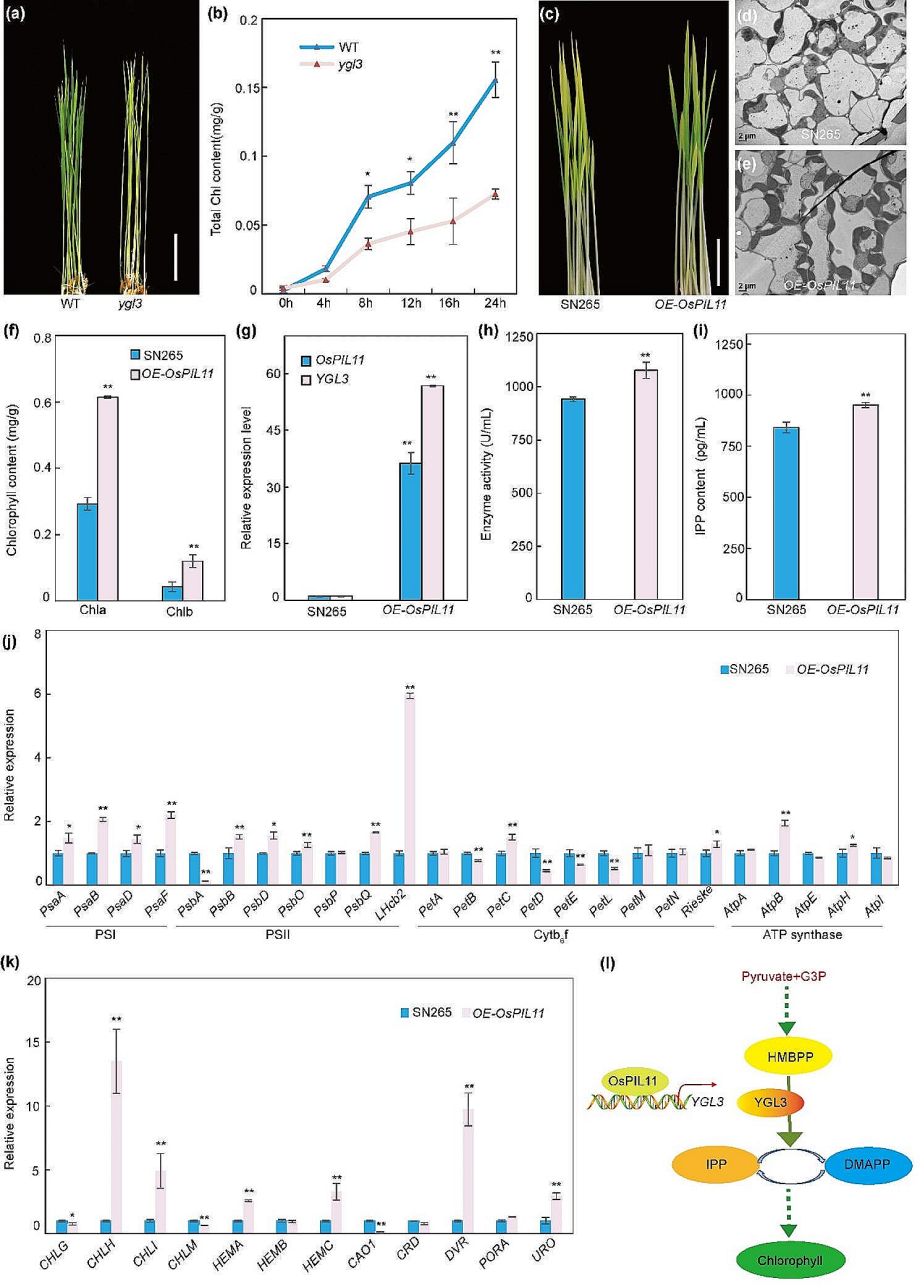
Expression and subcellular localization of OsPIL11. **(a)** Transactivation activity assay of OsPIL11 in yeast cells. **(b)** Subcellular localization of OsPIL11. GFP, green fluorescence; Red, D53 mCherry fluorescence. Scale bars = 10 μm. **(c)** Relative expressions of *OsPIL11* in various tissues based on qRT-PCR analysis (R, root; S, stem; L, leaf; LS, leaf sheath; P, panicle; Seed, developing endosperm at 6 d after fertilization)

Transactivation activity assay showed that OsPIL11^1–274aa^ had transcriptional activation activity in yeast cells (Fig. 7a). Meanwhile, yeast two hybrid and GST pull-down assay showed that OsPIL11 can form a homodimer (Fig. S9a, b). To determine the subcellular localization of OsPIL11, we performed a transient expression assay in rice protoplasts. OsPIL11-GFP fusion protein was localized in the nucleus and overlapped with the mCherry signal of D53-mCherry fusion protein, a known nuclear protein (Fig. 7b). We further explored the expression of *OsPIL11* by qRT-PCR. *OsPIL11* was expressed ubiquitously in all tissues, with the highest expression in the leaf (Fig. 7c).

### *OsPIL11* Positively Regulates Chlorophyll Synthesis via *YGL3* in the De-etiolation Process

Like other leaf-color mutants with defects in chlorophyll synthesis, the light-dependent synthesis of chlorophyll was also defective in the de-etiolation process in *ygl3* (Fig. 6a). To examine the rate of light-dependent chlorophyll biosynthesis, we grew WT and *ygl3* plants in darkness at 28 °C for 7 d and then exposed the plants to light. The rate of chlorophyll synthesis was significantly lower in *ygl3* that in the WT during the greening of etiolated seedlings (Fig. 6b). *OsPIL11* was the transcription activator of *YGL3*; however, whether *OsPIL11* affects chlorophyll synthesis during de-etiolation requires further study. Next, we generated overexpression (OE) plants of *OsPIL11* in the SN265 background. The expression level of *OsPIL11* was notably upregulated by approximately 36 times in the *OE-OsPIL11* plants (Fig. 6g). Compared to the SN265 plants, the *OE-OsPIL11* plants showed a higher greening rate and greater accumulation of chlorophyll during the de-etiolation process (Fig. 6c, f). Furthermore, the SN265 plants exhibited fewer chloroplasts compared to the *OE-OsPIL11* plants (Fig. 6d, e). Considering the role of OsPIL11 in the activation of *YGL3*, we evaluated the activity of 4-hydroxy-3-methylbut-2-enyl diphosphate reductase and the IPP contents in the SN265 and *OE-OsPIL11* plants. The enzymatic activity and IPP content were significantly higher in the *OE-OsPIL11* plants compared to in the SN265 plants (Fig. 6h, i). We also examined the expressions of genes related to thylakoid membrane and chlorophyll synthesis in the SN265 and *OE-OsPIL11* plants using qRT-PCR. Compared to SN265, the expression levels of most genes related to the thylakoid membrane and chlorophyll synthesis were upregulated in *OE-OsPIL11*; in contrast, the expressions of these genes were downregulated in *ygl3* (Figs. 4c and d and 6j and k). Collectively, these results indicate that *OsPIL11* regulates chlorophyll synthesis via *YGL3* during the de-etiolation process.

## Discussion

### *YGL3* Plays a Critical Role in Chloroplast Development

The MEP pathway is responsible for the production of essential isoprenoids in plants, which is essential for plant development (Bouvier et al. 2005). Recently, studies on the MEP pathway have increased rapidly. In *Arabidopsis*, the transfer DNA insertion of *ISPE* and *ISPF* impaired the development of chloroplasts and pigment biosynthesis (Hsieh and Goodman 2006; Hsieh et al. 2008). Cheng et al., and Huang et al., identified *ISPE* and *ISPF* in rice and demonstrated their involvement in regulating chloroplast development and chlorophyll biosynthesis (Chen et al. 2018; Huang et al. 2018). Likewise, a mutation in *CLA1*, the gene encoding 1-deoxy‐d‐xylulose 5‐phosphate synthase (DXS), affected chloroplast development and pigment biosynthesis in mutants, resulting in albino leaves (Araki et al. 2003). In the current study, we isolated *YGL3* from the EMS mutant population that encoded a thylakoid membrane-localized 4-hydroxy-3-methylbut-2-enyl diphosphate reductase (Fig. 3g, j). The mutation of *YGL3* resulted in yellow-green leaves, impaired chloroplast development, low chlorophyll accumulation, and poor photosynthetic efficiency, similar to the phenotype of *ISPH* (homologs of *YGL3*) mutation in *Arabidopis* (Hsieh and Goodman 2005) (Fig. 1; Fig. S1a). Chloroplast development was hindered by low temperature in temperature-sensitive mutants such as *v1*, *v2*, *v3*, *wsl5*, and *dua1* (Kusumi et al. 1997; Sugimoto et al. 2007; Yoo et al. 2009; Liu et al. 2018; Cui et al. 2019). Similarly, at 22 °C, the chloroplast ultrastructure and pigment contents were obviously abnormal in *ygl3*, and the abundance of YGL3 protein was affected by temperature (Figs. 1d, e, f, k and n and 3d; Fig. S2c, f), which suggesting that the activity of the YGL3 protein in *ygl3* mutant is temperature-sensitive. Interestingly, the expression of MEP pathway genes *zb7* and *DXS* has been shown to be regulated by temperature (Phillips et al. 2008; Lu et al. 2012). These data suggested that the expression of genes related to the MEP pathway may be regulated by temperature, and its molecular mechanism needs further exploration. The thylakoid membrane is an important component of chloroplasts, and the stability of its proteins complexes is crucial for chloroplast development. The protein contents and mRNA levels of thylakoid membrane protein complexes were notably lower in *ygl3* (Fig. 4a, d). On the other hand, the transcript levels of most genes associated with chlorophyll biosynthesis were dramatically reduced in *ygl3* compared to the WT (Fig. 4c). Taken together, these results demonstrate that *YGL3* plays a crucial role in chloroplast development and chlorophyll biosynthesis.

### The Mutation of *YGL3* Disturbs the MEP and MVA Pathways

In the cytoplasm, acetyl-CoA is required for the syntheses of IPP and DMAPP via the MVA pathway, which serve as precursors for sterols, sesquiterpenes, ubiquinone, dolichols, and other isoprenoids (Eisenreich et al. 2004). Pyruvate and glyceraldehyde 3-phosphate are required for the production of IPP and DMAPP through the MEP pathway in plastids, providing precursors for chlorophyll, phylloquinones, hormones, etc. (Bach et al. 1999). Several enzymes control the flux of the MEP pathway, and the overexpression of *DXS*, *DXR*, and *ISPG* in different organisms increases the formation of isoprenoid products products (Lois et al. 2000; Estevez et al. 2001; Mahmoud and Croteau 2001; Enfissi et al., 2005; Bertomeu et al. 2006; Paulet et al. 2006; Li et al. 2017). In this study, we found that *YGL3* encodes the final rate-limiting enzyme in the MEP pathway and exhibits 4-hydroxy-3-methylbut-2-enyl diphosphate reductase activity (Fig. 3h). The mutation of *YGL3* negatively affected the activity of 4-hydroxy-3-methylbut-2-enyl diphosphate reductase and reduced the content of IPP, a catalytic product of the MEP pathway (Fig. 3h, i; Fig. S8a, b). Both the IPP content and the 4-hydroxy-3-methylbut-2-enyl diphosphate reductase activity were significantly increased in the complementary transgenic plants compared to the WT (Fig. S8c). Based on an assay of mRNA levels, the expressions of most genes involved in the MEP pathway (e.g., *DXR*, *DXS*, *ISPF*, *ISPG*, and *YGL3*) were dramatically reduced in *ygl3* compared with the WT (Fig. 4b). We also examined the expressions of genes involved in the MVA pathway. The transcription levels of *HMGR1* and *HMGR2* genes were significantly up-regulated by the mutation of *YGL3* (Fig. 4b). HMGR catalyzes the conversion of 3-hydroxy-3-methylglutaryl-CoAto mevalonic acid, which has been identified as a rate-limiting enzyme (Vranova et al., 2013). Previous studies have indicated that crosstalk occurs between the MEP and MVA pathways (Kasahara et al. 2002; Bick and Lange 2003; Hemmerlin et al. 2003; Laule et al. 2003). These findings suggested that the disruption of the MEP pathway in the *ygl3* mutant leads to an imbalance in intracellular isoprenoids content, which may trigger feedback regulation of the MVA pathway on the disruption of the MEP pathway. In conclusion, these results suggest that mutation in *YGL3* severely disrupts both the MEP and MVA pathways.

### OsPIL11 Regulates Chlorophyll Biosynthesis during De-etiolation by Directly Activating *YGL3*

Accumulating evidence suggests that transcription factors play essential roles in regulating chlorophyll biosynthesis during seedling de-etiolation. Transcription factors bind with specific motifs in the promoters of specific sets of genes to regulate their functions. A double mutant of *FHY3* and *FAR1* exhibited reduced protochlorophyllide accumulation compared with the wild type, and chlorophyll biosynthesis was regulated by binding with the FBS (CACGCGC) motif in the *HEMB1* promoter (Tang et al. 2012). Zhong et al. reported that *EIN3* and *EIL1* activate the expressions of *PORA* and *PORB*, the key enzymes involved in the chlorophyll biosynthesis, inhibit photo-oxidative damage, and accelerate seedling de-etiolation in cooperation with PIF1 (Zhong et al. 2009). *AtPIF1* and *AtPIF3* control chloroplast development and chlorophyll biosynthesis by directly regulating the transcript levels of genes related to chlorophyll synthesis (Stephenson et al. 2009; Huq et al. 2004). Likewise, *OsPIL14* binds to the G-box in the promoter of *OsFLU1* to regulate chlorophyll synthesis in rice (Li et al. 2019). In the current study, we identified *OsPIL11*, a transcription factor in the bHLH family that encodes a nuclear protein with constitutive expression (Fig. 6b, c). Subsequently, based on in vitro and in vivo biochemical evidence, we demonstrated that OsPIL11 can directly bind to the promoter of *YGL3* and activate its transcription (Fig. 5b–e). *OsPIL11* positively regulated chlorophyll synthesis during seedling de-etiolation; the *OE-OsPIL11* plants exhibited increased leaf greening rate and greater chlorophyll accumulation compared to the SN265 plants (Fig. 7c, d, e). Moreover, the *OE-OsPIL11* plants had higher 4-hydroxy-3-methylbut-2-enyl diphosphate reductase activity and IPP content compared to the SN265 plants, indicating that OsPIL11 increased the efficiency of the MEP pathway by activating the expression of *YGL3* (Fig. 7h, i). Previous study demonstrated that chlorophyll accumulated upon light exposure (Toledo-Oritiz et al., 2014). As the synthesis of chlorophyll directly depend on the MEP pathway, the transcriptional regulation of MEP pathway genes can affect the accumulation of the chlorophyll, however, the molecular mechanisms for this is still unclear. Here our results showed that light-responsive transcription factor OsPIL11 interacted with MEP pathway coordinated regulate chlorophyll synthesis. Therefore, we proposed the following mechanism by which OsPIL11-*YGL3* regulates chlorophyll biosynthesis (Fig. 7l). *YGL3* encodes a 4-hydroxy-3-methylbut-2-enyl diphosphate reductase and regulates the chlorophyll content by controlling the syntheses of IPP and DMAPP. OsPIL11 directly activates the expression of *YGL3*, promoting the conversion of HMBPP to IPP and DMAPP with the subsequent accumulation of chlorophyll.

## Materials and methods

### Plant Materials and Field Management

The *ygl3* mutants were identified from the mutant library of ZHU 1 S (Z1S), a thermo-sensitive genic male sterile line of rice created by EMS mutagenesis. *Nipponbare* (NIP, *japonica* rice), D50 (tropical *japonica* rice), and 02428 (*japonica* rice) were crossed with *ygl3* to construct F_2_ populations for genetic analysis. All plants were cultivated in a paddy field in the summer or growth chambers following the standard agronomic practices and growing conditions at the China National Rice Research Institute in Hangzhou, China.

### Analyses of Photosynthetic Rate and Chlorophyll Content

The photosynthetic rates of the *ygl3* and WT plants (three biological replicates of each) were measured in the morning from 8:30–11:00 using a photosynthetic apparatus (Li-6800, Li-Cor, Lincoln, Nebraska, USA). Fresh leaves of the WT and *ygl3* plants at the seedling stage with the fourth leaf were used to determine the chlorophyll contents (Wu et al. 2007). The leaves were immersed in ethanol, incubated in the dark at 4℃ for 48 h, and then centrifuged. The absorption of the supernatant was measured at 665 and 649 nm using a DU800 spectrophotometer (Beckman Coulter, USA). Each test included three biological replicates.

### Microscopy

The chloroplast structures in the fourth leaf of the WT and *ygl3* mutant plants were observed by TEM. Leaves were cut into small pieces, fixed with 2.5% glutaraldehyde diluted with phosphate buffer (pH 7.2), and kept under vacuum until the specimens were immersed. The specimens were then fixed, dehydrated, embedded, sliced, and finally imaged by TEM (Hitachi H-7650, Tokyo, Japan) (Inada et al. 1998).

### Map-Based Cloning of *YGL3* Gene

To map *ygl3*, leaves from 896 plants with the mutant phenotype were selected from the *ygl3*/NIP F_2_ population. More than 156 polymorphic SSR markers evenly distributed on the entire genome were used for the linkage analysis of *ygl3*. SSR and InDel markers were developed according to the nucleotide polymorphisms between Z1S and NIP in the corresponding regions (Table S3). The candidate genes were amplified for sequencing from both *ygl3* and Z1S genomic DNA. The sequencing results were analyzed using SnapGene v.3.2.1.

### RNA Extraction and qRT-PCR Analysis

Total RNA was extracted from the root, stem, leaf, panicle, leaf sheath, and seed (5 and 10 DAF). Total RNA was extracted from the root, stem, leaf, panicle, leaf sheath, and seed (6, 9, 12, 15, and 21 DAF) using Trizol (Invitrogen, Carlsbad, USA). The RNA was reverse-transcribed into cDNA (ReverTra Ace qPCR RT Kit; Toyobo, Osaka, Japan) following the manufacturer’s protocol, and qRT-PCR was performed using SYBR Green RT-PCR Master Mix (Toyobo) and a LightCycler 480 system (Roche, Basel, Switzerland). The rice *Actin* gene (*Os03g0718150*) was used as an internal control. Gene expression was calculated using the 2^−ΔΔCT^ method. The sequences of primers used in this experiment are listed in Table S3.

### Plasmid Construction and Rice Transformation

To construct the complementation vector of *ygl3*, the WT *YGL3* genomic fragment from Z1S, including its promoter, was cloned into the binary vector pCAMBIA1300. The CRISPR/Cas9 targets of *YGL3* were cloned into BGK03 (Biogle, Hangzhou, China; http://www.biogle.cn/index/excrispr). To construct the OE vector of *OsPIL11*, the CDS sequence of *OsPIL11* was cloned into pCUBI1390. The plasmids were introduced into *Agrobacterium tumefaciens* strain EHA105. The complementation vector was transformed into *ygl3*. The OE vector and CRISPR/Cas9 vector were transformed into SN265 and NIP, respectively. All primers used for plasmid construction are listed in Table S3.

### Subcellular Localization

The coding sequences of *YGL3* and *OsPIL11* excluding the stop codons were cloned into pAN580 to generate the YGL3-GFP and OsPIL11-GFP constructs, respectively. The other two fragments of YGL3^1–31aa^ and YGL3^32–459aa^ were also constructed in pAN580. All vectors including the empty vector were transiently expressed in rice protoplasts using previously described protocols (Zhang et al. 2011). The GFP fluorescence signal was detected using a Zeiss LSM510 laser scanning confocal microscope (Karl Zeiss, Jena, Carl Zeiss AG, Germany).

### GUS Staining

The promoter region of *YGL3* (2 kb upstream of ATG) was cloned into pCAMBIA1305. The vector was transformed into NIP, and the positive T_1_ transgenic plants were then used to detect GUS activity according to the previously described protocol (Hull and Devic 1995).

### Protein Extraction and Western Blot Analysis

Total proteins were extracted from the leaves of the WT, *ygl3*, and transgenic plants using extraction medium (25 Mm Tris-HCl at pH 7.4, 150 Mm NaCl, 1 mM EDTA, 1% Nonidet P-40, 5% glycerol, 1 mM PMSF, and Roche protease inhibitor). The extracts were centrifuged at 14,000 rpm for 5 min at 4℃, and the supernatant was collected; this process was repeated three times. The proteins were separated by SDS-PAGE, transferred to polyvinylidene fluoride membranes, and immunoblotted with the corresponding antibodies. The control was β-actin.

### Analyses of 4-hydroxy-3-methylbut-2-enyl Diphosphate Reductase Activity and IPP Content

The 4-hydroxy-3-methylbut-2-enyl diphosphate reductase enzyme activity and IPP content were determined by ELISA. Fresh leaves of 15-day-old seedlings of WT and *ygl3* plants were chopped, weighed (0.1 g), extracted with PBS (pH 7.4), homogenized, and centrifuged for 20 min (3000 rpm, 4 °C). The supernatant was assayed using a 4-hydroxy-3-methylbut-2-enyl diphosphate reductase ELISA kit BE-E18918O1 (Jiangsu Boshen Biological Technology Co. Ltd, China). The absorbance values at 340 nm were measured with a microplate spectrophotometer (Infinite200 PRO; TECAN, Mannedorf, Switzerland). The IPP content was determined in a parallel assay using an ELISA kit (BS-E18975O1, Jiangsu Boshen Biological Technology Co. Ltd., China).

### Luciferase Transient Transcriptional Activity Assay in Rice Protoplasts

For the luciferase transient analysis, the *YGL3* promoter was constructed in the 190LUC vector. The cDNA sequence of *OsPIL11* was cloned into the None vector and used as an effector. The rLUC vector was used as an internal control. The relative luciferase activity ratio was calculated as the ratio of firefly luciferase (LUC) to *Renilla* luciferase (REN) using a dual-luciferase reporter assay system (E1910, Promega) (Zong et al. 2016). Rice protoplasts were prepared, and the plasmid was transformed according to the protocol of Zhang et al.

### Yeast One-Hybrid Assay

For yeast one-hybrid assay, the coding sequence of *OsPIL11* and the promoter sequence of *YGL3* were cloned into the pB42AD and pLacZi vectors, respectively. The plasmids were co-transformed into the EGY48 yeast strain, grown in SD/-Trp-Ura medium, and transferred onto an X-gal pate for interaction detection.

### Expression and Purification of Fusion Protein

The coding sequence of *OsPIL11* was cloned into the PGEX-4T-1 and Pmal-c2x vectors, and the fusion protein expressed by Rosetta Competent cells induced with 0.5 mmol isopropyl-1-thio-D-galactopyranoside at 26℃ for 12 h. GST and GST-OsPIL11 were purified using a GST recombinant protein purification kit (BBI, C600327-0001) according to the manufacturer’s instructions. MBP-OsPIL11 was purified using amylose resin (NEB, E8021S).

### EMSA

EMSA was carried out using a chemiluminescent EMSA kit (Beyotime, GS009). Oligonucleotide probes were synthesized and labeled with biotin (Sunya Biological Technology). Unlabeled probes were used as competitors. The probes were incubated with both GST and GST-OsPIL11. The protein–oligonucleotide complexes were separated on 6% non-denaturing polyacrylamide gel, and the protein–DNA signals were observed using a ChemiDoc^TM^MP Imaging System (Bio-Rad).

### Yeast Two-Hybrid Assay

The full-length, 1-274aa, 1-324aa, and 275-445aa coding sequences of OsPIL11 were cloned separately in PGBKT7. The coding region of OsPIL11 was also cloned in PGADT7. The clones were then transformed into the yeast strain Y2H. The yeast strains were grown on SD/-Leu-Trp medium, and the interactions were detected on SD/-Ade-His-Trp-Leu medium by adding X-α-Gal.

### In vitro GST Pull-Down Assay

The coding region of OsPIL11 was inserted into the PGEX4T-1 and Pmal-c2x vectors for fusion with the GST and MBP tags, respectively. After expression of the fusion protein by Rosetta Competent cells, GST and GST-OsPIL11 were incubated with MBP-OsPIL11 at 4℃ for 5 h, and glutathione high-capacity magnetic agarose beads (Sigma, G0924) were washed five times. MBP and GST fusion proteins were detected by MBP antibody (CW0288M) and GST antibody (AF0174), respectively.

## Conclusion

In this study, we identified *YGL3* as a positive regulator of rice chloroplast development. Mutation of *YGL3* exhibits yellow-green leaves, lower chlorophyll content and abnormal chloroplast ultrastructure. The 4-hydroxy-3-methylbut-2-enyl diphosphate reductase activity and IPP content were dramatically reduced in *ygl3* compared to the WT. Immunoblot analysis and qRT-PCR confirmed that chlorophyll syntheses genes and thylakoid membrane proteins genes were be regulated in the *ygl3* mutant. Furthermore, *OsPIL11* acts upstream of *YGL3* and regulate chlorophyll synthesis during the de-etiolation process by activate its expression. The findings provides a theoretical basis for understanding the molecular mechanisms by which the MEP pathway regulate chloroplast development in rice.

### Electronic Supplementary Material

Below is the link to the electronic supplementary material.

**Supplementary Material 1:** Supplemental Figures

**Supplementary Material 2: Table S1**. Analysis the genetic characteristic of *ygl3*

## Data Availability

The data sets supporting the results of this article are included within the article and its additional files.

## Abbreviations

*ygl3*: *yellow-green leaf 3*
MEP: Methylerythritol phosphate
MVA: Mevalonate
TEM: Transmission electron microscopy
IPP: Isopentenyl diphosphate
DMAPP: Dimethylallyl diphosphate. ORF:Open reading frame
NCBI: National center for biotechnology information
qRT-PCR: Quantitative real-time polymerase chain reaction
GUS: β-Glucuronidase
GFP: Green fluorescent protein
HMBPP: 4-hydroxy-3-methyl-but-2-enyl pyrophosphate
PSI: Photosystem I
PSII: Photosystem II, Cytb_6_f:cytochrome b6f complex
ATP synthase: Adenosine 5-triphosphate synthase
PIL: Phytochrome-interacting bHLH factors-LIKE
EMS: Ethylmethylsulfone
ELISA: Enzyme linked immunosorbent assay
EMSA: Electrophoretic mobility shift assay
