# Supplementary material for: YGL3 Encoding an IPP and DMAPP Synthase Interacts with OsPIL11 to Regulate Chloroplast Development in Rice
Source: Rice (N Y). 2024 Jan 17;17:8. doi: 10.1186/s12284-024-00687-y (PMC10792152; doi:10.1186/s12284-024-00687-y)
Supplement: Supplementary file 1 — Supplementary Material 1: Supplemental Figures [file 12284_2024_687_MOESM1_ESM.docx]

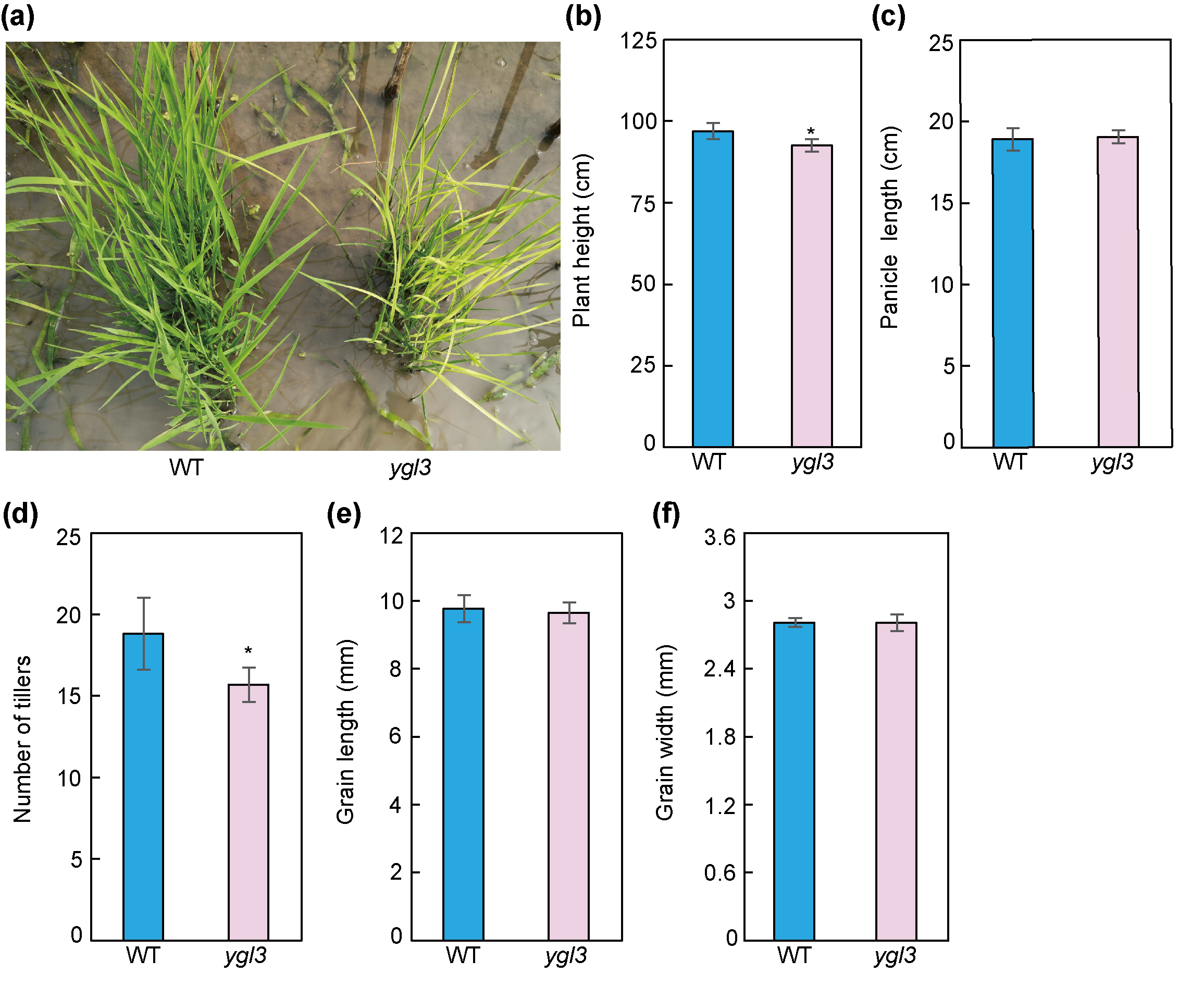


**Fig. S1 Major agronomic traits of the WT and *ygl3*. (a),** Phenotype of the WT and *ygl3* mutant at seeding stage**. (b),** Plant height. **(c),** Panicle length. **(d)**, Number of tillers. **(e)**, Grain length. **(f),** Grain width. Data are shown as mean ± SD from ten biological replicates. Asterisks indicate statistical significance as determined by Student’s *t*-test (***P*<0.01, 0.01<**P*<0.05).


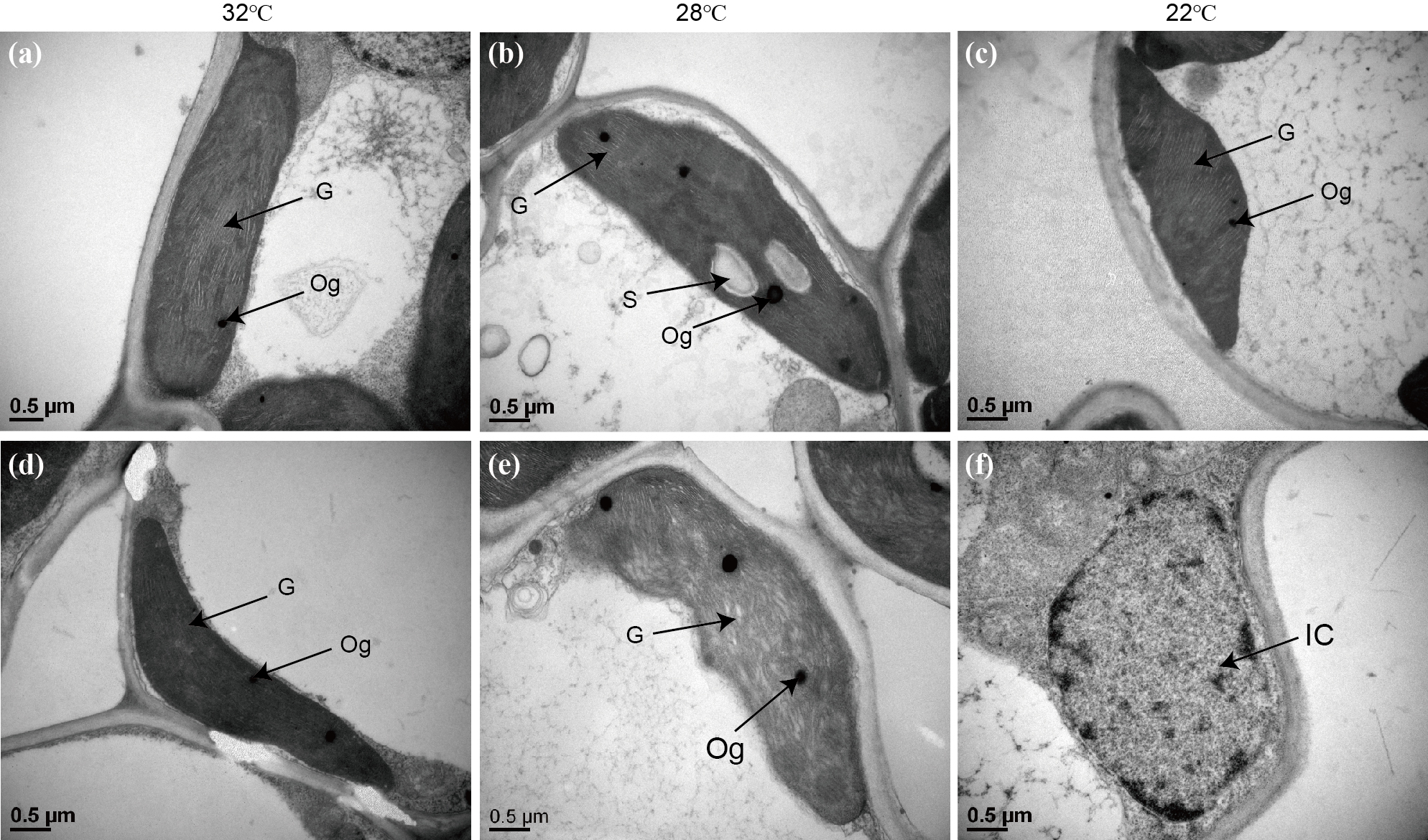


**Fig. S2 Chloroplast ultrastructure of the WT and *ygl3*.** **(a-f)**, Ultrastructure of chloroplast in cells of the leaves of two-week-old wild-type **(a, b, c)** and *ygl3* **(d, e, f)**. G, grana stacks; Og, osmiophilic plastoglobuli; S, starch granule; IC, immature chloroplast.


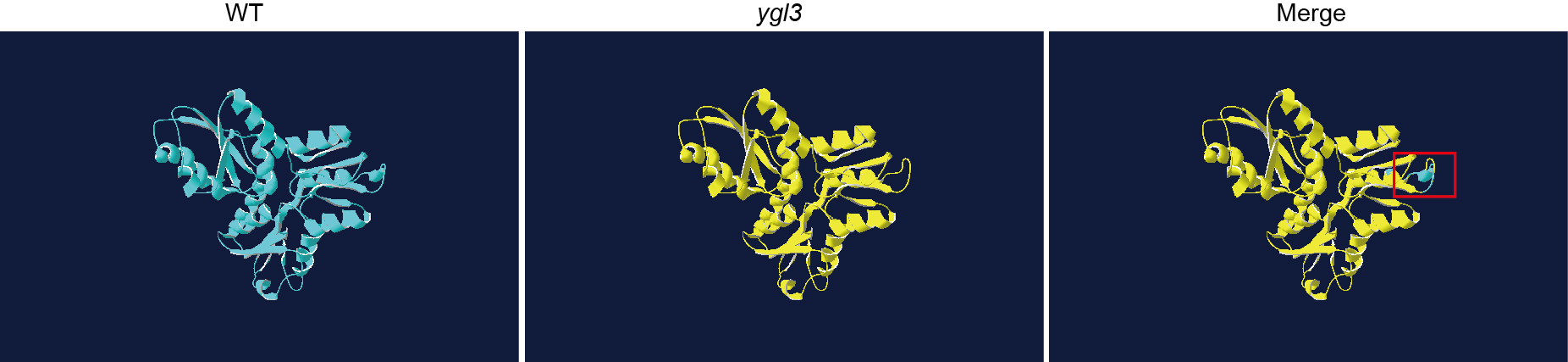


**Fig. S3** **Predicted 3D protein structures of YGL3 and ygl3.** The 3D structures of protein were predicted by Phyre2. The red box showed the region difference between WT and *ygl3*.


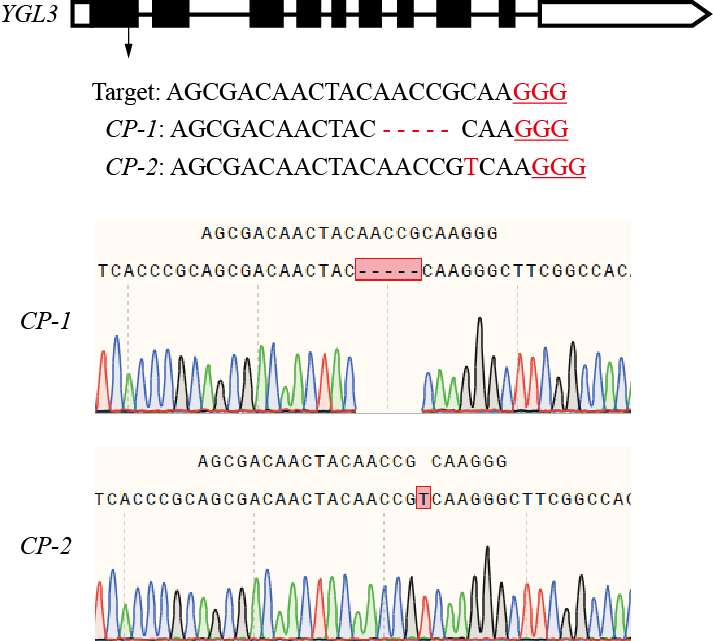


**Fig. S4** **Knockout mutations of *YGL3* by CRISPR/Cas9 system.** Exons and introns are represented by black boxes and lines, respectively. White boxes indicate untranslated regions.


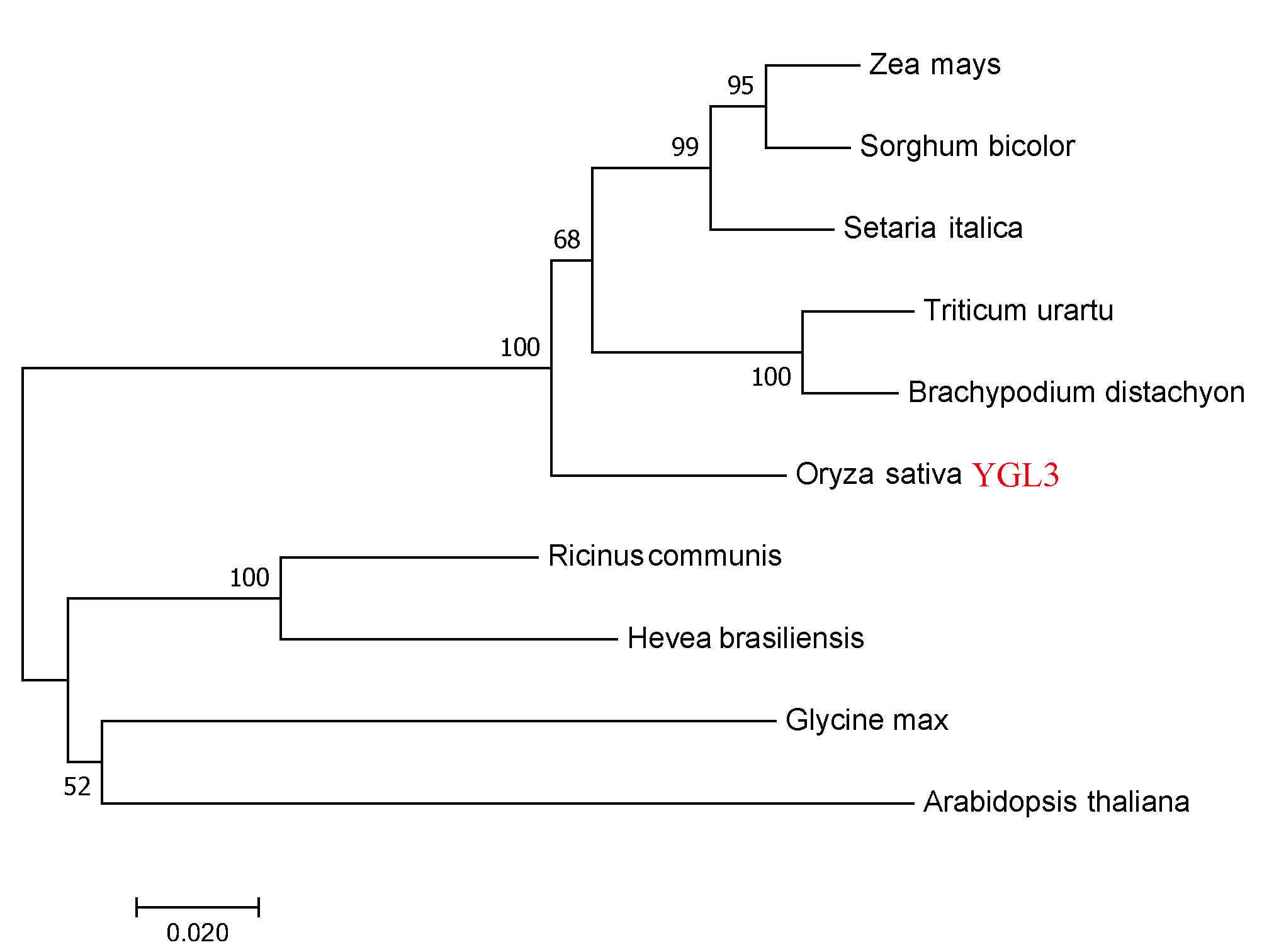


**Fig. S5** **Phylogenetic analysis of YGL3.** The phylogenetic tree was constructed by using MEGA 7.0.


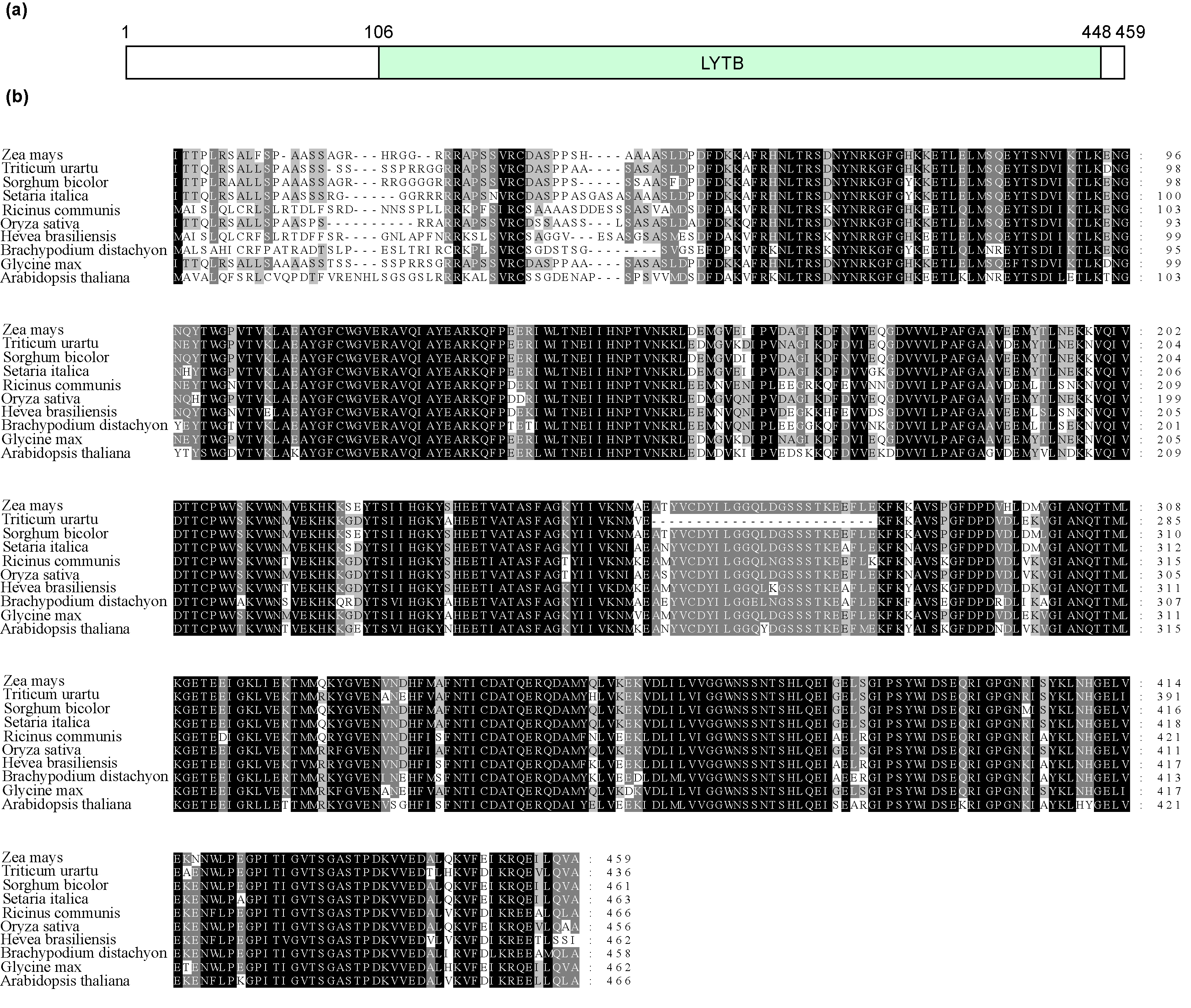


**Fig. S6** **YGL3 is a LYTB domain‐containing protein.** (a) YGL contains a LYTB domain. (b) Multiple sequence alignment of YGL3 and its homologs.


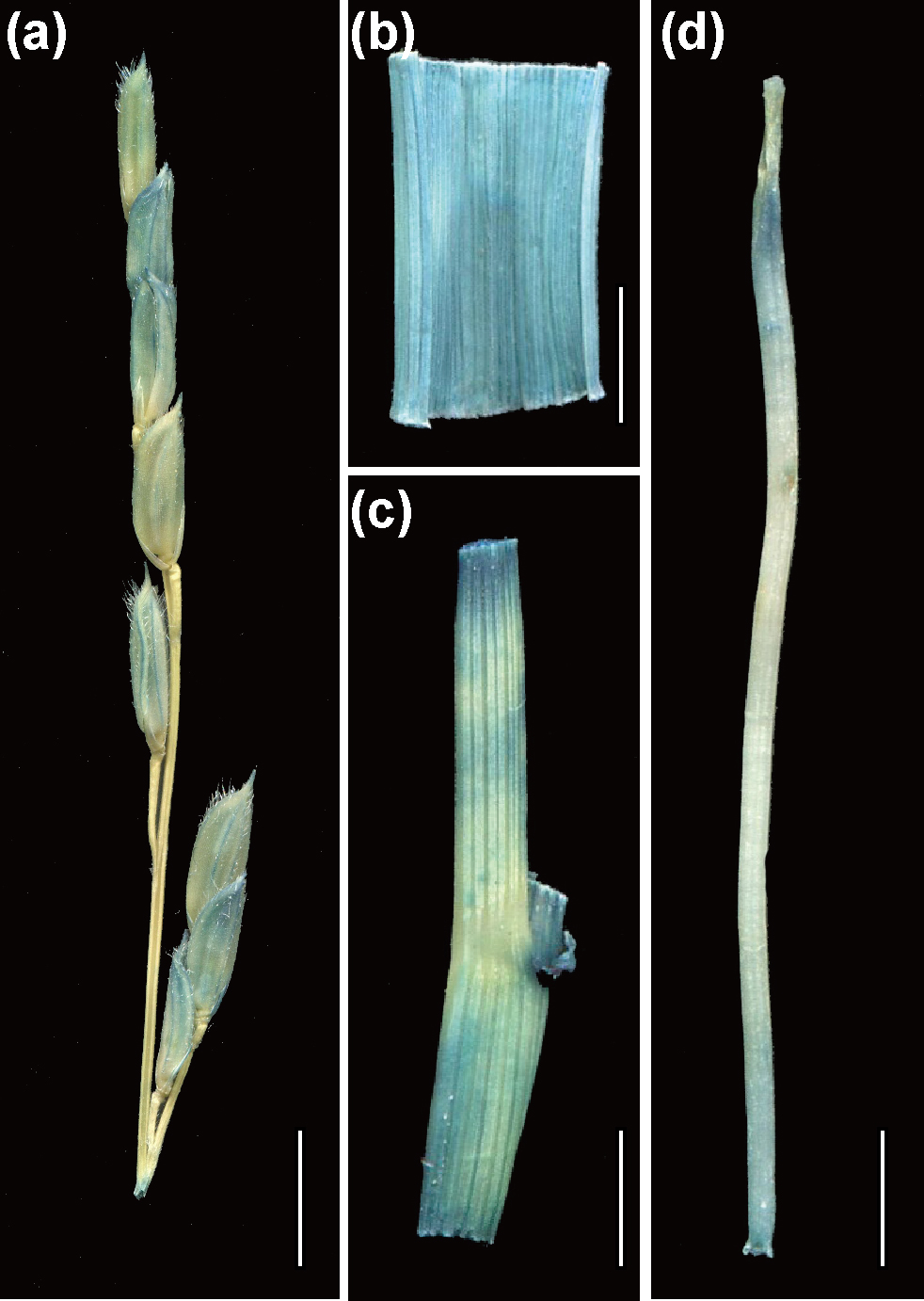


**Fig. S7** **GUS staining.** GUS expression in panicle **(a)**, leaf **(b)**, Leaf sheath **(c)**, and Root **(d)** driven by *YGL3* promoter. Bar=5 mm.


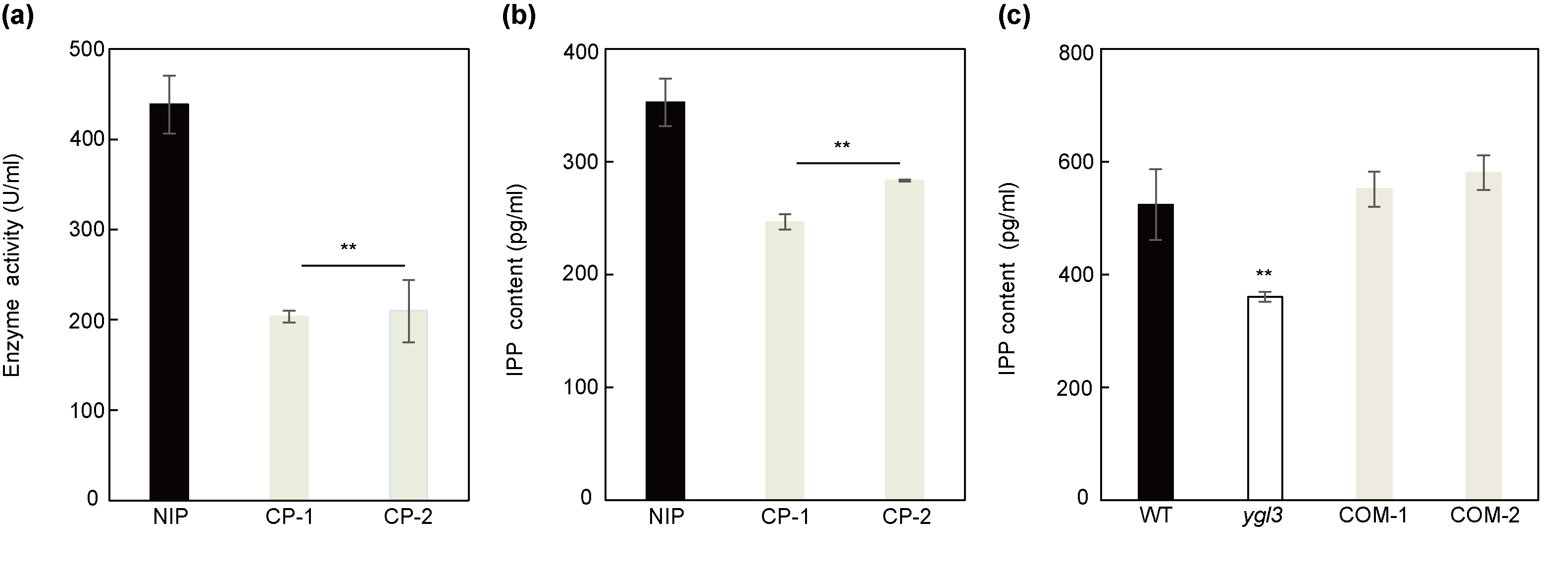


**Fig. S8** **The 4-hydroxy-3-methylbut-2-enyl diphosphate reductase activity and IPP content of transgenic plants. (a)**, The 4-hydroxy-3-methylbut-2-enyl diphosphate reductase activity in NIP and knockout transgenic plans. **(b and c)**, IPP content in knockout plants and complement plants, respectively. Data are shown as mean ± SD from three biological replicates. Asterisks indicate statistical significance as determined by Student’s *t*-test (***P*<0.01, 0.01<**P*<0.05).


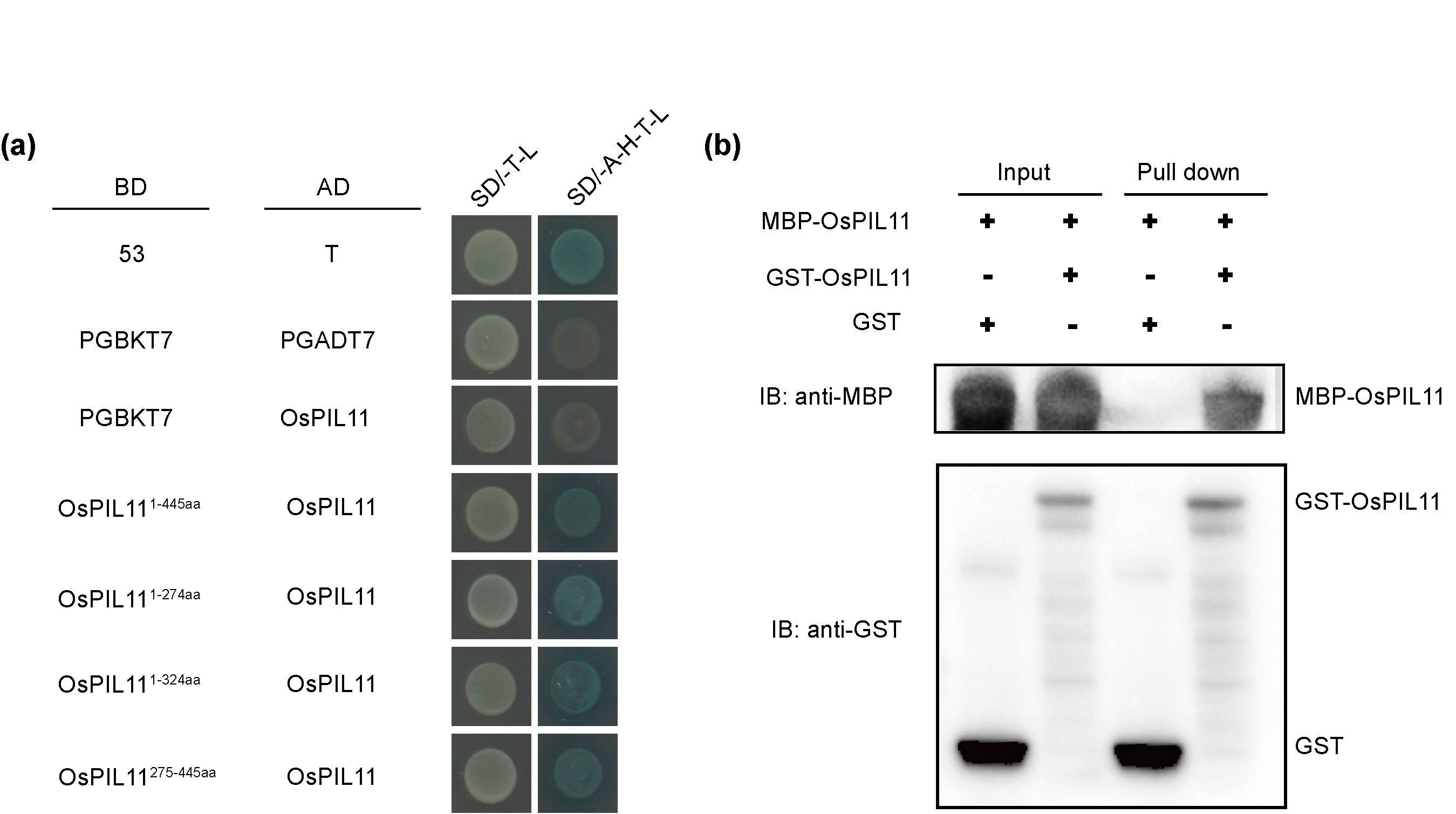


**Fig. S9 OsPIL11 forms a** [**homodimer**](javascript:;)**. (a)**, Yeast-two hybrid assay shows that OsPIL11 forms homologous complex. PGBKT7-53 and PGADT7-T as the positive control. The PGBKT7 and PGADT7 as the negative control. **(b)**, An *in vitro* GST pull-down assay.
